# An integrated metagenomics pipeline for strain profiling reveals novel patterns of bacterial transmission and biogeography

**DOI:** 10.1101/gr.201863.115

**Published:** 2016-11

**Authors:** Stephen Nayfach, Beltran Rodriguez-Mueller, Nandita Garud, Katherine S. Pollard

**Affiliations:** 1 Integrative Program in Quantitative Biology, University of California, San Francisco, San Francisco, California 94158, USA;; 2 Gladstone Institutes, San Francisco, California 94158, USA;; 3 Institute for Human Genetics, Institute for Computational Health Sciences, and Department of Epidemiology and Biostatistics, University of California, San Francisco, San Francisco, California 94158, USA

## Abstract

We present the Metagenomic Intra-species Diversity Analysis System (MIDAS), which is an integrated computational pipeline for quantifying bacterial species abundance and strain-level genomic variation, including gene content and single-nucleotide polymorphisms (SNPs), from shotgun metagenomes. Our method leverages a database of more than 30,000 bacterial reference genomes that we clustered into species groups. These cover the majority of abundant species in the human microbiome but only a small proportion of microbes in other environments, including soil and seawater. We applied MIDAS to stool metagenomes from 98 Swedish mothers and their infants over one year and used rare SNPs to track strains between hosts. Using this approach, we found that although species compositions of mothers and infants converged over time, strain-level similarity diverged. Specifically, early colonizing bacteria were often transmitted from an infant’s mother, while late colonizing bacteria were often transmitted from other sources in the environment and were enriched for spore-formation genes. We also applied MIDAS to 198 globally distributed marine metagenomes and used gene content to show that many prevalent bacterial species have population structure that correlates with geographic location. Strain-level genetic variants present in metagenomes clearly reveal extensive structure and dynamics that are obscured when data are analyzed at a coarser taxonomic resolution.

Microbial species play important roles in the different environments that they inhabit. However, different strains of the same species can differ significantly in their gene content (Greenblum et al. 2015; Zhu et al. 2015) and single-nucleotide polymorphisms (SNPs) (Schloissnig et al. 2013; Kashtan et al. 2014; Lieberman et al. 2014). These strain-level differences are important for understanding microbial evolution, adaptation, pathogenicity, and transmission. For example, strain-level differences have shed light on ecological differentiation of closely related bacteria (Shapiro et al. 2012), uncovered the presence of ancient subpopulations of marine bacteria (Kashtan et al. 2014), and highlighted extensive intra-species recombination (Snitkin et al. 2011; Rosen et al. 2015). Strain-level variation is also important for understanding microbial pathogenicity. Differences at the nucleotide level can lead to within-host adaptation of pathogens (Lieberman et al. 2014), and differences in gene content can confer drug resistance, convert a commensal bacterium into a pathogen (Snitkin et al. 2011), or lead to outbreaks of highly virulent strains (Rasko et al. 2011).

Metagenomic shotgun sequencing has the potential to shed light onto strain-level heterogeneity among bacterial genomes within and between microbial communities, yielding a genomic resolution not achievable by sequencing the 16S ribosomal RNA gene alone (Sunagawa et al. 2013) and circumventing the need for culture-based approaches. However, limitations of existing computational methods and reference databases have prevented most researchers from obtaining this level of resolution from metagenomic data. Assembly-free methods that map reads to reference genomes to estimate the relative abundance of known strains (Francis et al. 2013; Tu et al. 2014) are effective for well-characterized pathogens like *E. coli* that have thousands of sequenced genomes. However, such methods cannot detect strain-level variation for the vast majority of known species that currently have only a single sequenced representative. Other assembly-free approaches have been developed that use reads mapped to one or more reference genomes to identify SNPs (Schloissnig et al. 2013; Lieberman et al. 2014) and gene copy number variants (Greenblum et al. 2015; Zhu et al. 2015; Scholz et al. 2016) of microbial populations. These approaches have not been integrated together and/or made available as software. Recently, several software tools have been developed (Luo et al. 2015; Sahl et al. 2015) that use SNP patterns to phylogenetically type strains, but these methods do not capture the gene content of these organisms and may not be able to resolve strains in communities with high population heterogeneity. Additionally, existing methods do not provide comprehensive up-to-date genomic databases of bacterial species, thus limiting their utility across different environments. Assembly-based methods (Nielsen et al. 2014; Cleary et al. 2015) that seek to reconstruct microbial genomes without using reference genomes are a powerful alternative to assembly-free methods. However, these often require many samples, struggle to deconvolve closely related strains, or require manual inspection.

To address these issues, we developed the Metagenomic Intra-species Diversity Analysis System (MIDAS), which is a computational pipeline that quantifies bacterial species abundance and intra-species genomic variation from shotgun metagenomes. Our method integrates many features and leverages a comprehensive database of more than 30,000 reference genomes (for a comparison to existing methods, see Supplemental Table S1). Given a shotgun metagenome, MIDAS rapidly and automatically quantifies gene content and identifies SNPs in bacterial species, which is accurate for populations with a minimum of 1 and 10× sequencing coverage, respectively. These statistics enable quantitative analysis of bacterial populations within and between metagenomic samples.

To demonstrate the utility of this approach, we used MIDAS to conduct novel strain-level analyses on two data sets. First, we applied MIDAS to stool metagenomes from 98 Swedish mothers and their infants and used rare SNPs to track vertical transmission and temporal stability of strains in infants over the first year of life. Second, we used MIDAS to quantify gene content of prevalent bacterial species in 198 globally distributed marine metagenomes and identified significant intra-species population structure associated with geographic location and environmental variables. These analyses reveal striking microbial dynamics and structure that are missed when metagenomes are analyzed at a coarser taxonomic resolution.

## Results

### Identification of bacterial species with a consistent definition and efficient algorithm

**Figure 1.**
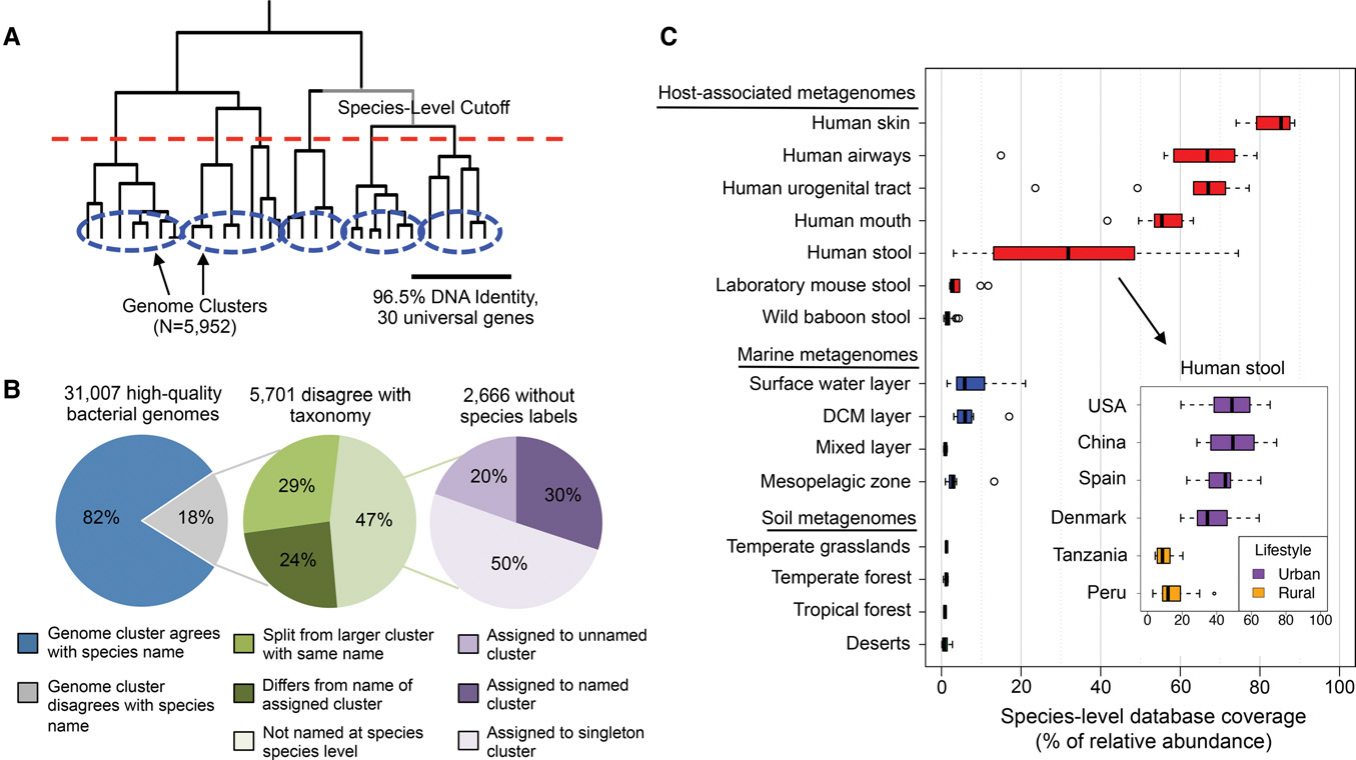
Construction of bacterial species database and its coverage of microbial communities across different environments. (*A*) In total, 31,007 genomes were hierarchically clustered based on the pairwise identity across a panel of 30 universal gene families. We identified 5952 species groups by applying a 96.5% nucleotide identity cutoff across universal genes, which is equivalent to 95% identity genome-wide. (*B*) Concordance of genome-cluster names and annotated species names. Of the 31,007 genomes assigned to a genome cluster, 5701 (18%) disagreed with the consensus PATRIC taxonomic label of the genome cluster. Most disagreements are due to genomes lacking annotation at the species level (47%). Other disagreements are because a genome was split from a larger cluster with the same name (29%) or assigned to a genome cluster with a different name (24%). (*C*) Coverage of the species database across metagenomes from host-associated, marine, and terrestrial environments. Coverage is defined as the percentage (0%–100%) of genomes from cellular organisms in a community that have a sequenced representative at the species level in the reference database. The *inset* shows the distribution of database coverage across human stool metagenomes from six countries and two host lifestyles.

To quantify strain-level genomic variation broadly and accurately, we built a comprehensive database of 31,007 high-quality bacterial reference genomes obtained from the Pathosystems Resource Integration Center (PATRIC) (Wattam et al. 2014). We accurately clustered these genomes into species groups to avoid inconsistent, erroneous, and incomplete annotations that afflict some microbial taxonomies (Mende et al. 2013) and to expand and improve upon previous efforts to systematically delineate bacterial species (Mende et al. 2013; Schloissnig et al. 2013; Varghese et al. 2015). Toward this goal, we hierarchically clustered reference genomes using the average pairwise percent identity across a panel of 30 universal genes (Fig. 1A) that we selected from a panel of 112 candidates (Supplemental Fig. S1; Supplemental Table S2; Wu et al. 2013). We found that the best gene families for identifying bacterial species were less conserved and more widely distributed across the tree of life relative to other genes we tested (Supplemental Fig. S2). For example, many ribosomal gene families were too conserved to differentiate closely related species (Supplemental Table S2). We applied a 96.5% nucleotide identity cutoff, which produced genome clusters that were highly concordant with a gold standard definition of prokaryotic species based on 95% genome-wide average nucleotide identity (Supplemental Table S3; Konstantinidis et al. 2006; Richter and Rossello-Mora 2009). Our procedure clustered the 31,007 bacterial genomes into 5952 genome clusters, representing distinct bacterial species (Supplemental Tables S4, S5). We inferred the phylogenetic relationships of these species using a concatenated alignment of the 30 marker genes (Supplemental Fig. S3). Because our algorithm uses a small set of highly informative marker genes, rather than genome-wide sequence comparisons, it will be efficient to update these genome clusters as additional genomes are sequenced.

The genome clusters we identified often differed from the PATRIC taxonomic labels (Fig. 1B). Our procedure clustered 2666 genomes (8.6% of total) that had not been previously annotated at the species level and reassigned species labels for 3035 genomes (9.8% of total) to either (1) group them with genomes that were not labeled as the same species in the reference taxonomy (*N* = 1380), or (2) split them from genomes with the same label in the reference taxonomy (*N* = 1655). Supporting our species definitions, we found that the bacterial species we identified tended to have distinct functional repertoires, with only 0.05% of FIGfam protein families (Meyer et al. 2009) shared between genomes from different species on average compared to >80% for pairs of genomes from the same species. In previous work, Mende et al. (2013) conducted a similar procedure to cluster genomes into species groups and found that the majority of disagreements with the NCBI taxonomy were supported by the literature.

### Current reference genomes cover the majority of human-associated bacterial species and highlight novel diversity in other environments

We evaluated how comprehensively our reference database covers the abundance of species present in different environments, as this is a requirement for conducting reference-based strain-level analyses. Previous work has shown large gaps in diversity between sequenced reference genomes and environmental microorganisms (Wu et al. 2009). To explore how well current genome sequences cover diversity present in metagenomes from various environments, we developed a novel approach that estimates the proportion of microbial genomes (including archaea and eukaryotes, but excluding viruses) in a metagenome that contain a sequenced representative at the species level in a reference database (Methods). This proportion, which we call “database coverage,” indicates the degree to which species in a sample are known versus novel.

We applied this method to stool metagenomes from the Human Microbiome Project (HMP) and four other studies of the human gut (Supplemental Table S6). We found that our reference database of 5952 bacterial species had high coverage of microbial communities from the human body (Fig. 1C). This included high database coverage of samples from the skin (mean = 83%), nasal cavity (mean = 63%), urogenital tract (mean = 62%), mouth (mean = 55%), and gastrointestinal tract (mean = 49%). The human gut communities with highest database coverage came from individuals in the United States, Europe, and China that live urban lifestyles, which is consistent with a previous report (Sunagawa et al. 2013). In contrast, gut microbiomes of individuals from Tanzania and Peru that live hunter-gatherer and agricultural lifestyles had much higher levels of novel species with no sequenced representative in our database. This finding extends the previous discoveries of elevated levels of novel genera (Schnorr et al. 2014) and functions (Rampelli et al. 2015) in the gut microbiome of African hunter-gatherers. Our analysis points to specific phylogenetic gaps in the set of currently sequenced bacterial genomes. Gut communities with lower database coverage tended to have higher levels of several genera including *Coprococcus*, *Subdoligranulum*, *Dorea*, and *Blautia*, whereas communities with higher database coverage tended to have higher levels of the genus *Bacteroides* (Supplemental Fig. S4). We conclude that there is a clear bias of genome sequencing to date toward species associated with hosts from industrialized countries.

In contrast to the human microbiome, a relatively small proportion of microbes present in other environments were captured by our reference database (Fig. 1C; Supplemental Table S6). This included very low coverage for stool metagenomes from laboratory mice (mean = 4.3%), which was surprising because mice are often used as a model system for studying the human microbiome. Coverage was also strikingly low in marine (means: surface water = 8.2%, deep chlorophyll maximum layer = 6.9%, subsurface epipelagic mixed layer = 1.0%, mesopelagic zone = 4.0%) and soil (means: desert = 1.0%, forest = 1.0%, grassland = 1.3%, tundra = 1.1%) environments. These estimates emphasize the massive gap that remains between the microbial diversity found in nonhuman environments and that represented by sequenced bacterial reference genomes. Strain-level analyses can still be performed for environments with low database coverage, but only for those species with sequenced representatives.

### An integrated pipeline for quantifying intra-species genomic variation from shotgun metagenomes

**Figure 2.**
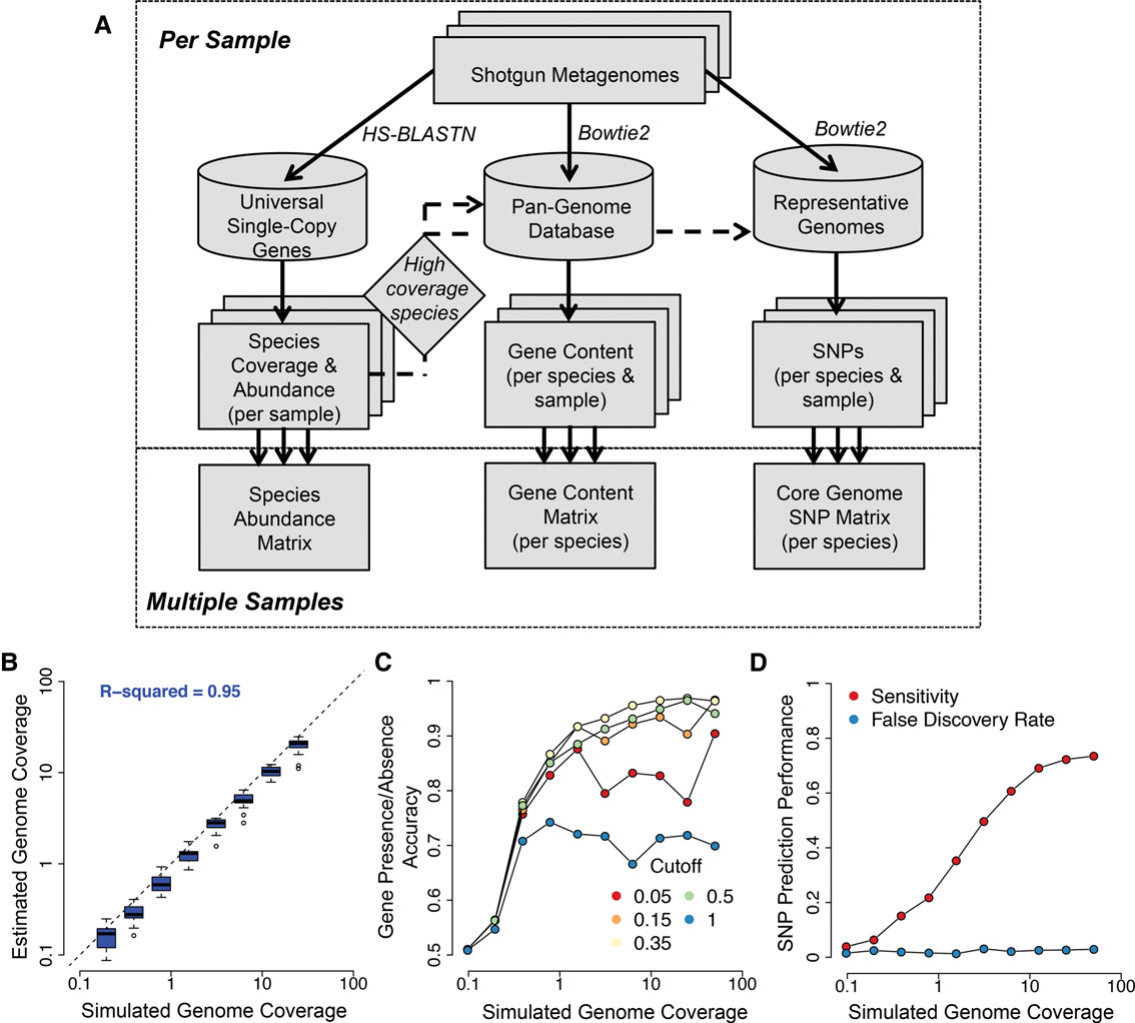
An integrated pipeline for profiling species abundance and strain-level genomic variation from metagenomes. (*A*) The MIDAS analysis pipeline. Reads are first aligned to a database of universal-single-copy genes to estimate species coverage and relative abundance per sample. For species with sufficient coverage, reads are next aligned to a pan-genome database of genes to estimate gene coverage, copy number, and presence–absence. Finally, reads are aligned to a representative genome database to detect SNPs in the core genome. The core genome is defined directly from the data by identifying high-coverage regions across multiple metagenomic samples. (*B–D*) To evaluate performance for each component of MIDAS, we analyzed 20 mock metagenomes composed of 100-bp Illumina reads from microbial genome-sequencing projects. Each community contained 20 organisms with exponentially decreasing relative abundance. We tested the ability of MIDAS to estimate species coverage and to predict genes and SNPs present in the strains of the mock communities compared to the reference gene and genome databases. (*B*) Species coverage is accurately estimated. Each boxplot indicates the distribution of estimated genome coverages across 20 mock communities for the top eight most abundant species out of 20 analyzed. (*C*) Gene presence–absence is accurately predicted when genome coverage is above 1×, and a gene copy number cutoff of 0.35 is used. Accuracy = (Sensitivity + Specificity)/2; Sensitivity = (number of genes correctly predicted as present)/(number of total genes present); Specificity = (number of genes correctly predicted as absent)/(number of total genes absent). (*D*) SNPs are detected with a low false-discovery rate and good sensitivity when genome coverage is above 10×. Sensitivity = (number of correctly called SNPs)/(number of total SNPs); False Discovery Rate = (number of incorrectly called SNPs)/(number of called SNPs).

We next developed MIDAS, which is a software tool that processes shotgun metagenomes to sensitively and automatically quantify species abundance and strain-level genomic variation for any of the bacterial species in our database (Methods; Fig. 2A). MIDAS was designed to be fast, memory efficient, and to scale with the rapid increase in sequenced reference genomes (Supplemental Fig. S5). Using a single CPU, MIDAS processes approximately 5000 reads per second and requires ∼3 gigabytes of RAM.

MIDAS first estimates the coverage and relative abundance of bacterial species by mapping reads to a database of universal single-copy gene families (Supplemental Table S7). Species are automatically identified with sufficient coverage for gene content and SNP analyses directly from the shotgun metagenome. This enables population-genetic analysis of metagenomes without any prior knowledge about a community's composition. Additionally, this step prevents unnecessary, time-consuming alignments to genes and genomes from sequenced organisms that are not present in a community.

To quantify the gene content of individual species in each metagenome, MIDAS maps reads to a pan-genome database. This database contains the set of nonredundant genes across all sequenced genomes from each species. It is generated on the fly to include only the subset of species with high sequencing coverage at universal single-copy genes in the metagenome being analyzed. The coverages of genes in the pan-genome database are normalized by the coverage of the universal single-copy gene families, yielding an estimated copy number of a gene per cell of a given species in each sample. Additionally, copy numbers are thresholded to predict gene presence–absence per sample.

To identify SNPs of individual species, MIDAS maps reads to a genome database. This database contains one representative genome sequence per species, and it only includes species with high sequencing coverage at universal single-copy genes in the metagenome being analyzed. Representative genomes are selected to maximize their sequence identity to all other genomes within the species. The core genome of each species is identified directly from the data using nucleotide positions in the representative genome that are at high coverage across multiple metagenomic samples (Supplemental Fig. S6). SNPs are quantified along the entire core genome, including at sites that are variable between samples, but fixed within individual samples. Core-genome SNPs are useful because they occur in all strains of a species and facilitate comparative analyses.

MIDAS was validated using 20 mock metagenomes that we created by pooling Illumina reads from completed genome sequencing projects (Methods; Supplemental Tables S8, S9). These libraries are expected to contain sequencing errors and other experimental artifacts found in real short-read sequencing data that might prevent accurate estimation of species abundance and strain-level genomic variation. Using these data, we found that MIDAS accurately estimated the relative abundance of bacterial species (*r*^2^= 0.95), but slightly underestimated sequencing coverage (Fig. 2B). MIDAS accurately predicted the presence or absence of genes in species present with at least 1–3× sequencing coverage (Fig. 2C). Prediction accuracy was maximized at 0.96 for strains with greater than 3× coverage when using a threshold equal to 0.35× the coverage of universal single-copy genes—lower thresholds resulted in lower specificity, and higher thresholds resulted in lower sensitivity. MIDAS also called SNPs at a low false-discovery rate, but required between 5× and 10× coverage to identify the majority of SNPs present (Fig. 2D).

### Species and strain-resolved analyses shed light on vertical transmission of human gut microbiota

We hypothesized that the large numbers of SNPs that MIDAS can identify from individual metagenomes could be leveraged to detect bacterial strains unique to a host and transmission of strains between hosts. More specifically, we believed this approach could be used to shed light on the extent and timing of mother-to-infant (i.e., vertical) transmission of gut bacteria. An understanding of vertical transmission is critical for determining the extent to which the microbiome—and by extension microbiome-mediated phenotypes—are inherited. Recent studies have found significant overlap in species between mothers and their infants over the first year of life (Backhed et al. 2015; Bokulich et al. 2016), and many microbial taxa that are heritable (Goodrich et al. 2016). These studies did not examine whether strains are vertically transmitted, and recent work has shown that species-level analyses alone can be insufficient to resolve transmission events (Li et al. 2016). Mother-to-infant transmission of specific taxa has been resolved using culture-based techniques (Tannock et al. 1990; Martín et al. 2012; Makino et al. 2013; Milani et al. 2015), but it is not clear whether these results are generalizable across microbiome species. Other studies have examined the development of the infant gut microbiome (Koenig et al. 2011), including at the strain level (Luo et al. 2015; Yassour et al. 2016), but did not assess vertical transmission. Thus, the extent and timescale of vertical transmission and the stability of transmitted stains are currently not well established.

**Figure 3.**
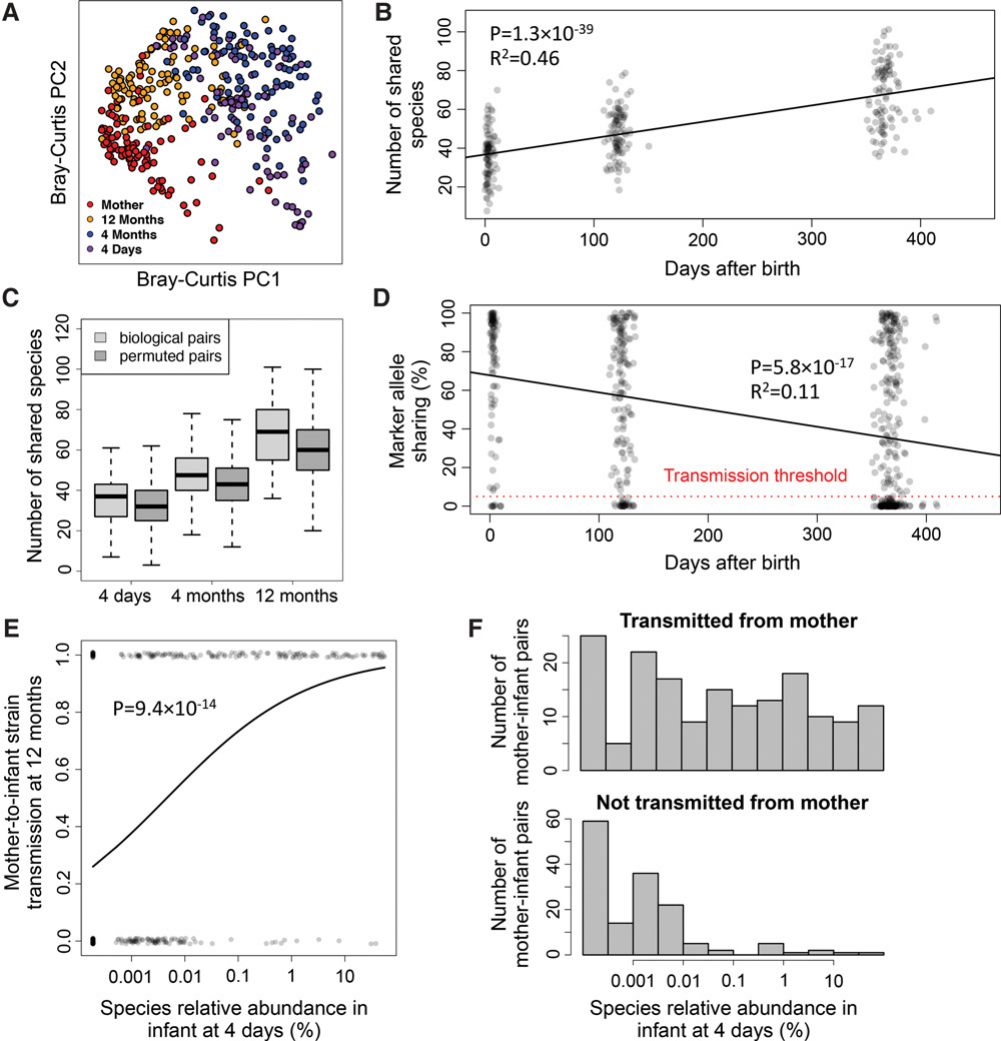
An increase in shared species but a decrease in shared strains over time between stool metagenomes from mothers and their infants. (*A*) Principal coordinate analysis of Bray-Curtis dissimilarity between species relative abundance profiles of stool samples from mothers and infants at 4 d, 4 mo, and 12 mo following birth. Species composition of infant microbiomes is most similar to mothers at 12 mo. (*B*) The number of shared species increases over time between mothers and their own infants. (*C*) This pattern for biological mother–infant pairs is similar to that of unrelated mothers and infants (permuted pairs). (*D*) In contrast, marker allele sharing decreases over time between mothers and their infants for shared species with greater than 10× sequencing coverage, indicating highest strain similarity at 4 d. Allele sharing is defined as the percentage of marker alleles in the mother that are found in the infant. The horizontal red dotted line indicates the 5% marker allele threshold used for defining vertical transmission events. (*E*) Early colonizing species are transmitted vertically, whereas late colonizing species are not. The horizontal axis indicates the relative abundance of bacterial species at 4 d. The vertical axis indicates whether a strain of the species was transmitted from the mother (*y* = 1) or not (*y* = 0) at 12 mo. The curve is a logistic regression fitted to data points. (*F*) Histograms indicate the distribution of relative abundance at 4 d for strains that were transmitted and not transmitted from an infant's mother.

To quantify strain transmission from mother to infant, we applied MIDAS to the Backhed and colleagues stool metagenomes from 98 mothers and their infants at 4 d, 4 mo, and 12 mo after birth (Backhed et al. 2015). We found that bacterial species alpha diversity was lowest in newborns and increased over time, species beta diversity was highest in newborns and decreased over time, and samples clustered by host age based on Bray-Curtis dissimilarity between species relative abundance profiles (Fig. 3A; Supplemental Fig. S7). Compared to infants, mothers had more diverse microbiomes that tended to harbor more unshared (i.e., unique to host) species (77% versus 48%, *t*-test *P* < 2.2 × 10^−16^). Despite this, we found a large number of shared species between infants and their mothers, which increased over time as diversity increased in the infants (Fig. 3B). These species-level trends agree with the results of the original study that used different methods to identify species (Backhed et al. 2015). Surprisingly, we found nearly as many shared species between permuted mother–infant pairs in which vertical transmission did not occur (Fig. 3C), suggesting that increased similarity of species in a mother and her infant over its first year is unlikely the result of direct transmission.

To detect transmission of gut microbiota from mother to infant with high specificity and sensitivity, we developed a novel approach that uses SNPs output by MIDAS (Methods). First we identified species shared between mothers and their infants with greater than 10× sequencing coverage, which is required for sensitive detection of SNPs (Fig. 2D). Next, we identified rare SNPs within these species that were private to strains found in a mother and her infant. We refer to these SNPs as “marker alleles” because they serve as a marker for individual strains. To detect whether a transmission has occurred for a species, we quantified the percent of marker alleles found in a mother that were shared with her infant.

To validate that marker alleles could be used to track strains between hosts, we applied our method to stool metagenomes of healthy adults from the HMP (Methods). As a positive control, we compared marker alleles of species between metagenomes from the same individual at the same time point (technical replicates); as a negative control, we compared marker alleles of species between metagenomes from different unrelated individuals (nonreplicates). As expected, we found high allele sharing (mean = 79.5%) among technical replicates and low allele sharing among nonreplicates (mean = 1.01%) (Supplemental Fig. S8). The fact that allele sharing was <100% in the technical replicates and >0% in the nonreplicates likely results from a combination of factors, including read sampling variation, small sample sizes, and sequencing errors. For example, marker alleles may be found in other individuals when sample sizes are increased. To define a transmission event, we selected a marker allele sharing cutoff of 5%, which clearly separated the positive and negative controls (sensitivity = 99.8%; specificity = 96.6%). High sensitivity and specificity was consistently observed across species we tested (Supplemental Table S10).

**Figure 4.**
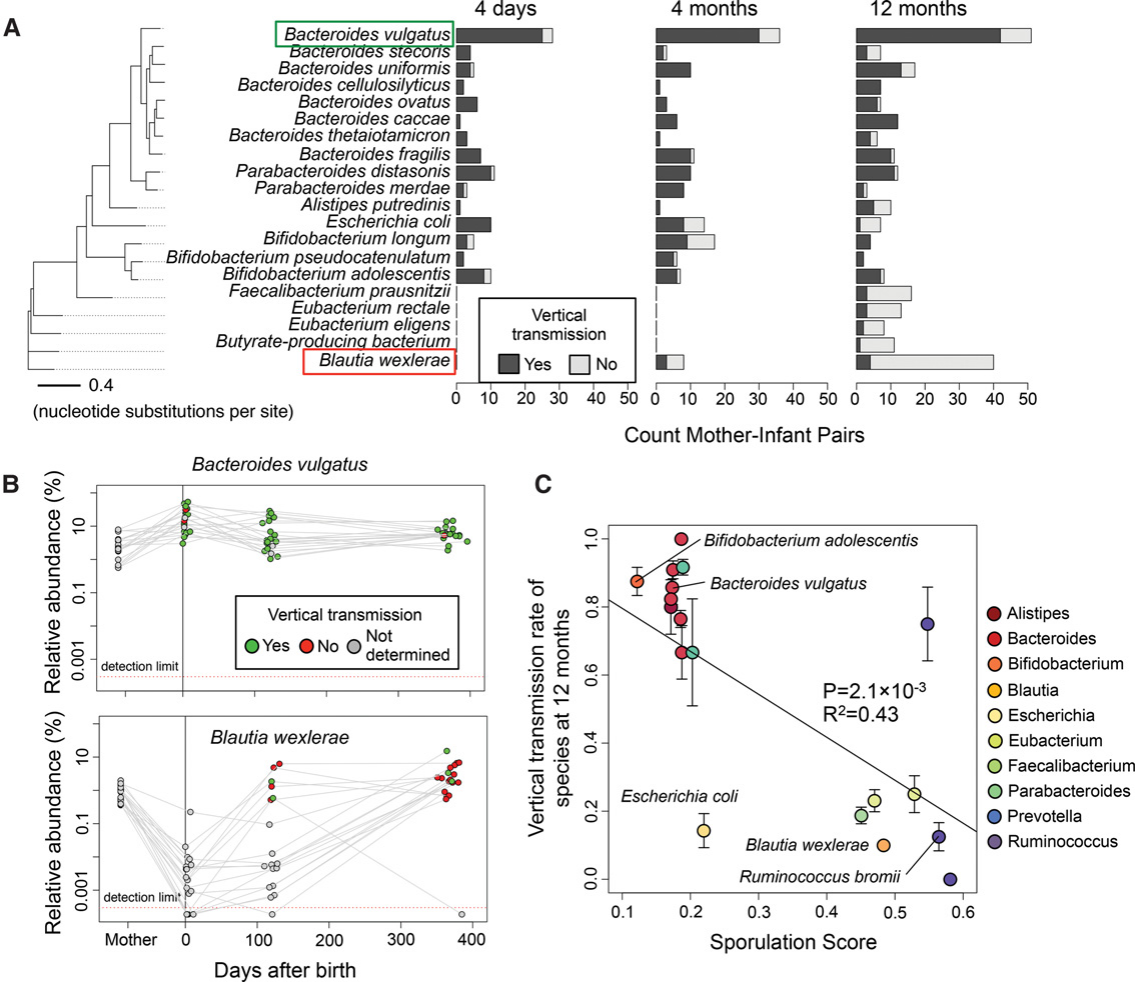
Distinct timing and vertical transmission patterns for microbiome species. (*A*) Vertical transmissions for bacterial species across mother–infant pairs at three time points. The 20 species with the greatest number of high-coverage mother–infant pairs are shown. A vertical transmission is defined as >5% marker allele sharing between mother and infant. The phylogenetic tree is constructed based on a concatenated DNA alignment of 30 universal genes (Supplemental Fig. S3) and shows that phylogenetically related species have similar transmission patterns. (*B*) *Bacteroides vulgatus* is an early colonizing species that is frequently transmitted vertically, whereas *Blautia wexlerae* is a late colonizing species that is rarely transmitted vertically. Gray points indicate there was insufficient sequencing coverage to quantify SNPs and determine transmission. (*C*) Species with low vertical transmission rates are predicted to be spore-formers with the ability to survive in the environment. Sporulation scores are genomic signatures of sporulation based on 66 genes (Browne et al. 2016). Error bars indicate one standard error in each direction. Only species with sporulation scores computed by Browne et al. (2016) and with three or more mother–infant pairs at 12 mo are shown.

Strikingly, we found that marker alleles were commonly shared between mothers and infants 4 d after birth (Fig. 3D). On average, 72% of marker alleles present in mother strains were found in newborns, which was only slightly less than the level of allele sharing observed from our positive control. Furthermore, of the 111 high-coverage species present in mothers and newborns, 101 (91%) had >5% marker allele sharing, indicating extensive vertical transmission of gut microbiota shortly after birth. Commonly transmitted species included *Bacteroides vulgatus* (25/28 mother–infant pairs with >5% marker allele sharing), *Parabacteroides distasonis* (10/11), *Bifidobacterium adolescentis* (8/10), and *Escherichia coli* (10/10) (Fig. 4A). There were no species present with greater than 10× coverage in 15 C-section-born infants and their mothers to assess transmission in these individuals. This likely reflects lower vertical transmission of the mother's gut microbes, but we cannot directly test that hypothesis with the available data.

Although we detected high strain similarity 4 d after birth, mother and infant strains significantly differed over time. Comparing strain-level SNPs in 4-mo and 12-mo infants to their mothers, we observed a sharp decrease in marker allele sharing and transmission rates (Figs. 3D, 4A). Across all species, transmission rates decreased from 91% at 4 d (101/111 shared species with >5% marker allele sharing), to 80% at 4 mo (131/163), and to 55% at 12 mo (172/313). C-section-born infants tended to have fewer vertically transmitted strains compared to vaginally born infants at 4 mo (χ^2^ *P* = 5 × 10^−8^, 3/14 versus 128/149 shared species with >5% marker allele sharing) and to a lesser extent at 12 mo (χ^2^ *P* = 0.06, 13/34 versus 159/279). This trend was in stark contrast to what we observed at the species level, in which there was an increase in the number of shared species over time and an increase in species-level compositional similarity. Thus, although the species-level composition of mothers and infants converged over time, the strain-level composition actually diverged.

We hypothesized that transmission rates decreased over time due to late colonization of the infant gut by new strains from the environment. If this were the case, then we would expect that (1) infant strains that were distinct from the mother at 12 mo had low abundance in the infant at earlier stages, and (2) strains transmitted from the mother at 4 d persisted in the infant gut over one year. Supporting our hypothesis, we found that the abundance of a species at 4 d was predictive of whether the strain of that species was transmitted from the mother (Fig. 3E,F; Supplemental Fig. S9). Specifically, strains of species with low abundance at 4 d but high abundance at 12 mo, like *Blautia wexlerae*, tended to be distinct from strains found in the mother. In contrast, strains of species with high abundance at 4 d and high abundance at 12 mo, like *Bacteroides vulgatus*, were similar to strains found in the mother (Fig. 4A,B). Also supporting our hypothesis, we found that the vast majority of strains that were transmitted from the mother at 4 d persisted in the infants at 4 mo (49/54 mother–infant pairs with >5% marker allele sharing) and at 12 mo (47/51).

Because the mother's stool was only sequenced at 4 d after birth, we cannot rule out the possibility that late colonizing strains came from the mother's gut but were not detected at the time of initial sampling. To address this issue, we quantified the temporal stability of strains in 157 healthy adults from the HMP over a time period of 300–400 d (Supplemental Fig. S10). We found high marker allele sharing (mean = 77.0%) and “transmission rates” (96.2%), which suggest that maternal strains may be quite stable over time, in agreement with previous work (Faith et al. 2013; Schloissnig et al. 2013). Together, our results suggest that bacteria are transmitted from mother to infant at birth, but bacteria from the environment increasingly colonize the infant gut over time.

If 12-mo-old infants were colonized by strains transmitted from the environment, then we would expect these bacteria to have the ability to form spores to protect them from ambient oxygen and survive outside of the host. To investigate this, we obtained a genomic signature of sporulation for many of the bacterial species found in mother–infant pairs from a recent study (Browne et al. 2016). The sporulation score was based on 66 genes in which values greater than 0.4 indicate true spore-formers. Strikingly, we found that species with low vertical transmission rates tended to have high sporulation scores, which supports the hypothesis that these bacteria are transmitted to the infants from the environment (*P* = 0.0013) (Fig. 4C). In contrast, bacterial species with high vertical transmission rates had low sporulation scores, suggesting that these species may be transmitted via direct physical contact. One of the exceptions to this pattern was the facultative anaerobe *Escherichia coli*, which was vertically transmitted early but transmitted from a different source later (Fig. 4A). To broadly characterize these patterns, we found the class Bacteroidia was enriched in vertical transmission events (χ^2^ *P* = 2.6 × 10^−18^), whereas the class Clostridia was depleted (χ^2^ *P* = 1.4 × 10^−22^) (Supplemental Table S11). These results highlight differences in the inheritance of gut microbiota that may be linked to distinct modes and timing of transmission between hosts.

### Global strain-level geography of prevalent marine bacteria

**Figure 5.**
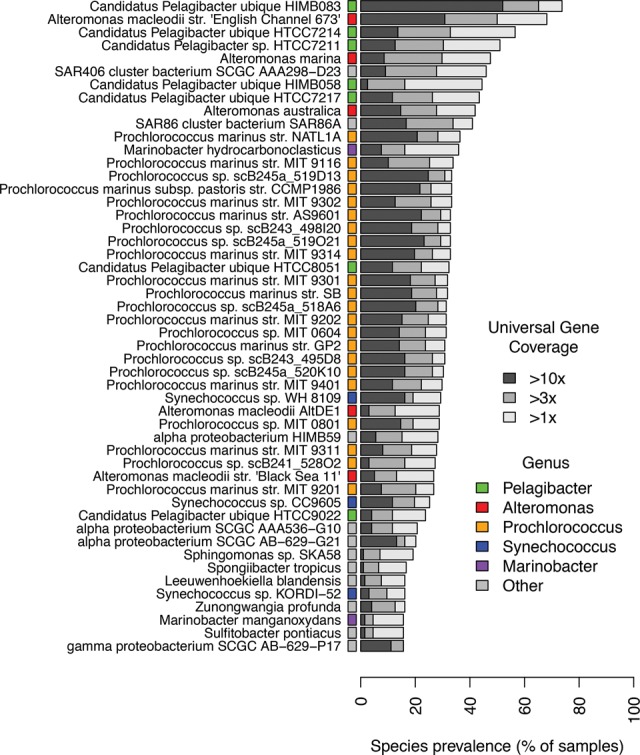
Prevalent bacterial species surveyed by the *Tara* Oceans expedition. Prevalence of 50 bacterial species across 198 ocean metagenomes. Latin names of species are indicated on the vertical axis. In cases in which multiple species had the same Latin name, the full name of the representative genome is shown. Many marine species have sufficient sequencing depth and prevalence for population-genetic analyses.

Many bacterial species are distributed widely across the world's oceans (Sunagawa et al. 2015). Yet genomes of a given species sampled near each other can differ significantly in their gene content (Kashtan et al. 2014). To explore the extent of population structure across different marine bacterial species on a global scale, we used MIDAS to quantify pan-genome gene content for prevalent species in 198 marine metagenomes from 66 stations along the *Tara* Oceans expedition (Supplemental Table S12; Sunagawa et al. 2015). Because we found that our database had relatively low coverage of the cellular organisms present in ocean samples (Fig. 1C), we first estimated relative abundance and coverage of bacterial species in each metagenome to identify marine species in which gene content could be reliably estimated (i.e., coverage greater than 3× across a high percentage of samples) (Fig. 5). Among these species were several members of the genera *Pelagibacter*, *Alteromonas*, *Synechococcus*, and *Marinobacter,* a large group of closely related *Prochlorococcus* species, and several unnamed *Alphproteobacteria* species. Reference pan-genome sizes for these species ranged from 1047 to 1311 genes in the streamlined genomes of SAR406 and SAR86 (each with one genome) to 6427 genes in the largest *Prochlorococcus* genome cluster (*N* = 26 genomes) and 7819 genes for *Alteromonas macleodii* (*N* = 4 genomes).

We discovered extensive variability of gene content for these prevalent species across the ocean metagenomes (Supplemental Table S13). Across all species, we found an average of 318 genes that differed between samples, ranging from 144 genes in *SAR86* to 700 in *Alteromonas marina*. We next quantified the percentage of genes that were different between samples using the Jaccard index and found that on average 19% of genes differed between samples. This level of genomic variability was higher than the 13% recently reported for human gut communities (Zhu et al. 2015), although this may be due to methodological differences. Regardless, our estimate of 19% is almost certainly an underestimate of the true level of gene content variation between populations, because MIDAS cannot measure the variation of genes that are present in strains but absent from sequenced reference genomes.

**Figure 6.**
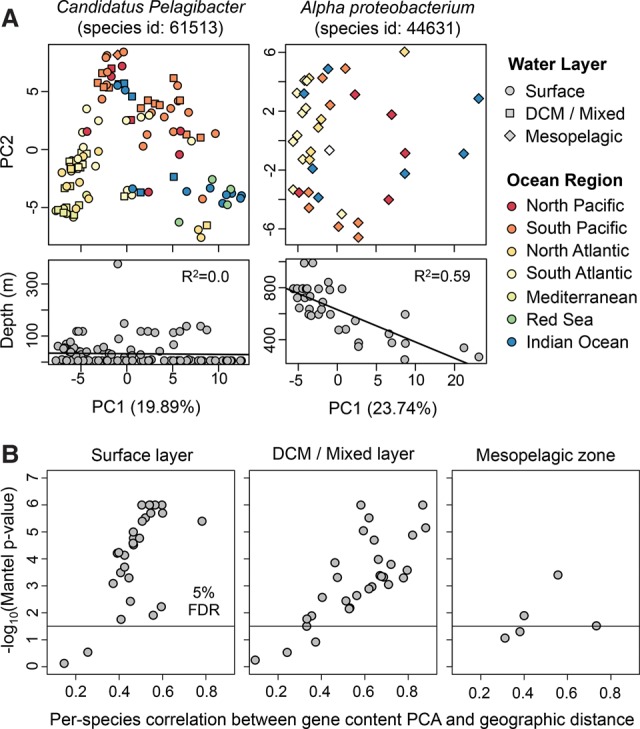
Gene content and geography are correlated for many marine bacteria. (*A*) Principal component analysis (PCA) of gene content for two bacterial species. Each point indicates a bacterial population from a different seawater sample. Point color and shape indicate the marine region and water layer, respectively. *Candidatus Pelagibacter* populations tend to cluster together based on ocean region, not ocean depth. In contrast, *Alpha proteobacterium* populations tend to cluster together based on ocean depth, not ocean region. (*B*) Gene content PCA and geographic distance are significantly correlated for most prevalent marine species. PCA distance was calculated using the Euclidian distance between PC1 and PC2 of the gene presence–absence matrix. Geographic distance was calculated using the great-circle distance between sampling locations. For each species, the correlation of these two distances (horizontal axis) and associated *P*-value (vertical axis) were computed using the Mantel test with 1 million permutations. Only one metagenome per location was included in the tests. The population structure of marine bacteria, based on the first two principal components of gene content, is correlated with geography for many species of bacteria.

To explore how this variation correlated with geography and sampling depth, we conducted a principal component analysis (PCA) of gene content for each bacterial species, as has been done to study the geographic structure of human populations using polymorphism data (Novembre et al. 2008). Strikingly, we found that the populations of many species clustered together by ocean region based on the first two principal components of gene content, regardless of sampling depth (Fig. 6A). For example, populations of one *Pelagibacter* species formed three discrete clusters corresponding to the Mediterranean Sea, South Atlantic Ocean, and South Pacific Ocean, and each cluster contained samples from multiple water layers. Similar results were obtained for many other species (Supplemental Fig. S11). Furthermore, we found that the population structure of the marine bacteria examined was highly consistent, regardless of the percent identity threshold used for defining pan-genome gene families (75%–99% identity) (Supplemental Figs. S12, S13).

To evaluate the extent of gene content biogeography across species, we computed the correlation between PCA distances and geographic distances (Methods) and found significant distance-decay in gene content for the majority of species tested (Fig. 6B). Furthermore, this pattern was observed both in samples from the surface water layer and the deep chlorophyll maximum layer—the majority of species we examined were not found in the mesopelagic water layer. A previous study found season to be a major driver of biodiversity patterns in the global ocean (Ladau et al. 2013). To explore whether season or other environmental variables were associated with strain-level population structure, we compared correlations of the first principal component of gene content (PC1) with geography and environmental variables (Supplemental Fig. S14). For 20/30 species tested, longitude (17/30) or latitude (3/30) was the strongest predictor of gene content, and each explained a significant proportion of gene content variation (22% and 8% on average). In contrast, day length (an indicator of season) explained less variation (4% on average) and was the most predictive covariate for only one *Prochlorococcus* species.

A few species showed relatively little geographic structure. Instead they had gene content variation that correlated with depth or marine layer. The most striking example of this was an unnamed *Alphproteobacteria* species which contained two genomes in our database obtained via single-cell sequencing (Stepanauskas 2012). This species was predominantly found in the mesopelagic layer (below 200 m) and increased in relative abundance with decreasing depth (Supplemental Fig. S15). Looking only at mesopelagic samples, we found that the first principal component of gene content (PC1) was strongly correlated with depth (*R*^2^= 0.59), suggesting little mixing of strains across depth. When we included samples from all marine layers, we found that samples from the mesopelagic and epipelagic zone formed separate clusters based on gene content, and there was still a strong correlation between PC1 and depth (*R*^2^= 0.57) (Supplemental Fig. S15). Our results could indicate that the populations at different depths contain genes for adaptation to the range of temperatures and nutrients across which this species is found. Supporting this hypothesis, we found hundreds of functions and pathways with gene copy numbers that were significantly correlated with depth (Supplemental Table S14).

Together, our results expand upon and even contradict patterns of marine bacterial biogeography observed at the species level. In particular, gene content analysis reveals that abundant and prevalent species are not ubiquitous at the strain level. Instead they show significant structure across geographic regions.

## Discussion

We developed MIDAS, an integrated computational pipeline that quantifies bacterial strain-level gene content and SNPs, as well as species abundance, from shotgun metagenomes. By coupling fast taxonomic profiling via a panel of universal-single-copy genes with sensitive pan-genome and whole-genome alignment, MIDAS can efficiently and automatically compare hundreds of metagenomes to more than 30,000 reference genomes to identify genetic variants present in the strains of each sample. Our publicly available software and data resources will enable researchers to conduct large-scale population-genetic analysis of metagenomes.

This first version of MIDAS has several limitations*.* Because it currently relies on bacterial reference genomes, MIDAS cannot quantify variation for novel species, novel genes, or known species from other groups of microbes (e.g., archaea, eukaryotes, and viruses). To accurately quantify strain-level gene content and SNPs, MIDAS requires greater than 1× and 10× sequencing coverage, respectively. This biases analyses toward the most abundant and prevalent species in an environment. MIDAS was nonetheless able to capture the majority of microbial species abundance across human body sites, making it well suited for uncovering strain-level variation of human-associated bacteria. In contrast, other environments appeared to be dominated by microbes missing from our reference database. For this reason, it will be important to update the database as the number (Land et al. 2015) and diversity (Wu et al. 2009) of microbial reference genomes continues to rapidly grow, as new experimental (Rinke et al. 2013) and computational (Nielsen et al. 2014) approaches uncover genome sequences of uncultured microbes. It will also be useful to incorporate genomes from other domains of life. Based on the design of our database and algorithm, MIDAS should scale with this growth of reference data.

To illustrate the utility of MIDAS, we analyzed stool metagenomes from a recently published study of 98 mothers and their infants over one year (Backhed et al. 2015) and used rare SNPs to track transmission of strains between hosts. Based on this analysis, we found extensive vertical transmission of early colonizing bacteria, which largely persisted in the infant for one year. Although significant attention has been paid to transmission of *Bifidobacterium* spp. (Martín et al. 2012; Makino et al. 2013; Milani et al. 2015), we found high transmission rates for many *Bacteroides* spp. We also found that late colonizing bacteria, including *Blautia*, *Ruminococcus*, *Eubacterium*, and *Facelibacerium,* were rarely transmitted from the mother. Instead the mother was colonized by a different strain of these species. Comparing these species to a recent study of sporulation in the human gut (Browne et al. 2016), we found that late colonizers tended to be spore-formers capable of surviving in the environment, whereas early colonizers were non-spore-formers. Together, these results suggest that only certain taxonomic groups of bacteria may be vertically inherited, whereas others are acquired from the environment. Our results build upon previous infant microbiome studies (Koenig et al. 2011; Backhed et al. 2015; Bokulich et al. 2016; Yassour et al. 2016) by showing that early and late colonizing species likely derive from different sources, which may be linked with their ability to form spores and survive in the environment. When the same metagenomes were analyzed at the species level, these patterns of transmission were missed, and a false signal of increasing transmission over time was detected due to convergence of the infant microbiome toward a more diverse and adult-like species profile. We conclude that species sharing frequently does not reflect direct transmission.

The bacterial taxa that tended to be transmitted vertically in our analysis differ from the taxa whose abundances were found to be heritable in a previous study of UK twins (Goodrich et al. 2016). For example, we estimated low vertical transmission for strains of *Blautia*, but Goodrich et al. (2016) found that the abundance of *Blautia* is highly heritable. Conversely, we estimated high vertical transmission for strains of *Bacteroides*, but this was one of the genera whose abundance was least heritable in the UK twins. Heritability does not require vertical transmission if related individuals are colonized by similar taxa that they acquire from the environment. On the other hand, the abundance of vertically transmitted taxa may not be heritable for extremely common taxa such as *Bacteroides*. Alternatively, strains vertically transmitted at birth could be lost as the infant ages, although we found them to be mostly retained over the first year of life.

Our analysis of mother–infant strain sharing leaves a few questions unanswered. One intriguing issue is the source of the strains that colonize the infant but are not present in the mother's stool microbiome at 4 d after birth. It is possible that some strains colonize the mother's gut later in the year and are then passed along to the infant, although this is unlikely based on the temporal stability of strains in the adult microbiome. The new strains could also derive from other sites on the mother's body, such as skin and breast milk, other people, food, or the environment. One caveat of our analysis is that we did not distinguish which strains were transmitted to the infant from the mother in cases in which mothers harbored multiple strains. Instead, we treated the transmission events as binary, whereby a transmission was defined as at least one strain being transmitted. It would be interesting to explore transmission as a quantitative variable in future work, including elucidating how the strain composition and genetic diversity of bacterial populations change as they are passed from mother to offspring and potentially undergo bottlenecks and selection.

To explore bacterial population structure using gene content, we applied MIDAS to metagenomes from the *Tara* Oceans expedition. We found a number of prevalent and abundant bacterial species, which shows that our method can be applied to different environments, despite low database coverage. Based on these results, we found that the gene content of many species in the epipelagic water layer (0–200 m) was structured geographically. This contrasts with previous work at the species level, which found that depth and temperature were the strongest predictors of community structure (Sunagawa et al. 2015). However, the gene content of other species found in the mesopelagic layer (200–1000 m) was structured by depth. As more genomes are sequenced from marine ecosystems, it should be possible to determine how generalizable these patterns are. Additionally, future work is needed to understand the extent to which these gene-level patterns are driven by adaptation to different environments in the ocean or are attributable to neutral processes, like genetic drift and/or migration.

Microbiome research is in an era in which metagenome-wide analyses can now pinpoint individual strains and genes that differ in presence or abundance between samples. Importantly, this level of resolution is not only revealing associations that are missed by analyses conducted at coarser taxonomic levels, but also patterns that oppose those inferred from species abundance distributions. A striking example is our discovery that infants share more gut bacterial strains with their mothers at birth than later in their first year of life, despite the fact that the species composition of their microbiomes becomes more similar as the infant ages. Without conducting a strain-level genomic analysis, one might incorrectly infer that vertical transmission of the gut microbiome is constant or increasing during the first year of life. Similarly, the high level of gene content variation that we observe in *Tara* Oceans bacteria and its strong correlation with geography in surface waters emphasizes functionally important differences in strains across global oceans that are missed when metagenomes are analyzed at the species level. It is clear that additional genome sequencing of environmental microbes is critical to advance these types of strain-level analyses in the future. Our analysis of database coverage points to specific environments and phylogenetic groups that are highest priority these efforts.

## Methods

### Sequence-based identification of bacterial species

We developed a procedure to cluster bacterial genomes into species groups based on the pairwise percent identity across a set of universal gene families, which was inspired by previous work (Mende et al. 2013). We began with 33,252 prokaryotic genomes downloaded from PATRIC (Wattam et al. 2014) in March 2015. Next, we used HMMER3 (Eddy 2011) with an *E*-value threshold ≤1 × 10^−5^ to identify protein homologs of 112 bacterial universal gene families (Wu et al. 2013) across the genomes. The HMMER3 search took too long for two gene families (B000042, B000044), which were dropped. When there were multiple homologs of a gene family identified in a genome, we took the homolog with the lowest *E*-value. We filtered out low quality genomes with fewer than 100 universal genes identified (*N* = 1837) or with more than 1000 contigs (*N* = 618), which left 31,007 high-quality genomes (Supplemental Table S5).

Next, we used BLASTN (Altschul et al. 1990) to perform sequence alignment of each gene family among all high-quality genomes. We filtered out local alignments in which either the query or target was covered by <70% of its length. We converted percent identities to distances using the formula *D*_*ab*_ = (100 − *P*_*ab*_)/100, in which *P*_*ab*_ was the percent identity of a gene between genomes *a* and *b*. This resulted in an undirected graph for each marker gene family in which nodes were genomes and edges were distances. We performed average-linkage hierarchical clustering for each graph using the program MC-UPGMA (Loewenstein et al. 2008). The output of MC-UPGMA is a tree, which we cut at different distance thresholds (0.01–0.10). Each cut of the tree yielded a set of genome clusters.

For validation, we compared each set of genome clusters to average nucleotide identity (ANI), which is considered to be a gold standard for delineating prokaryotic species (Konstantinidis et al. 2006; Richter and Rossello-Mora 2009), but was too computationally intensive to compute for all genome pairs. Specifically, we used the procedure described by Richter and Rossello-Mora (2009) to compute ANI for more than 18,000 genome pairs and labeled pairs of genomes with ANI ≥95% as members of the same species and pairs of genomes with ANI <95% as members of different species. We compared these labels to our genome clusters and classified each genome pair into one of the following categories: True positive: a clustered genome pair with ANI ≥95%; False positive: a clustered genome pair with ANI <95%; False negative: a split genome pair with ANI ≥95%; True negative: a split genome pair with ANI <95%. Using these classifications, we calculated the true positive rate (TPR), precision (PPV), and F1-score for each set of genome clusters corresponding to 90%–99% identity between pairs of genomes for a given marker gene (Supplemental Table S2).

Based on this evaluation, we identified a subset of 30 gene families that produced genome clusters that were in agreement with ANI, all with maximum F1-score >0.93 across thresholds. To increase clustering performance, we took the average pairwise distances across these 30 gene families and used these new distances to recluster genomes using MC-UPGMA (Supplemental Table S3). We found that a distance cutoff of 0.035 (96.5% nucleotide identity) maximized the F1-score at 0.98 and resulted in 5952 genome clusters (Supplemental Table S3). Each genome cluster was annotated by the most common PATRIC Latin name within the cluster (Supplemental Table S4).

### Genomic database construction

The clusters of genomes that corresponded to bacterial species were leveraged to compile a comprehensive genomic data resource used by MIDAS. First, we identified a representative genome from each species to use for detecting core-genome SNPs. Each representative genome was chosen to maximize its average nucleotide identity at the 30 universal genes (Supplemental Table S2) to other members of the species. Next, we built a database of 15 universal single-copy gene families (Supplemental Table S7) to use for estimating the abundance of the species from a shotgun metagenome. Gene families were selected based on their ability to accurately recruit metagenomic reads as well as being universal and single copy. The 30 gene families used for clustering genomes and the 15 gene families used for quantifying species abundance were in some cases different because of different selection criteria. Next, we used USEARCH (Edgar 2010) to identify the set of unique genes at 99% identity across all genomes within each species, which are used by MIDAS for metagenomic pan-genome profiling. This procedure clustered 116,978,184 genes from the 31,007 genomes into 31,840,245 gene families. We further clustered these genes at different levels of sequence identity (75%–95% DNA identity) to identity de novo gene families of varying size and diversity for downstream analyses. Functional annotations for all genes were obtained from PATRIC and include FIGfams (Meyer et al. 2009), Gene Ontology (Ashburner et al. 2000), and KEGG Pathways (Kanehisa and Goto 2000).

### Species abundance estimation

MIDAS uses reads mapped to 15 universal single-copy gene families to estimate the abundance of the 5952 bacterial species from a shotgun metagenome. These 15 gene families were selected from a set of 112 phylogenetically informative bacterial gene families (Wu et al. 2013) for their ability to accurately recruit metagenomic reads to the correct species. To evaluate how informative different gene families are for estimation of abundance, we simulated 100 100-bp reads from each of the 112 gene families in each of the 5952 species and used HS-BLASTN (Chen et al. 2015) to map these reads back to a database that contained the full-length gene sequences. To simulate the presence of novel species and strains, we discarded alignments between reads and reference sequences from the same genome. Each read was assigned to a species based on its top hit. Recruitment performance was measured using the F1-score. Based on this experiment, we identified 15 universal single-copy gene families that were best able to accurately assign the species from which metagenomic reads derived. Additionally, we identified the optimal percent identity cutoffs for mapping reads to the database, which ranged from 94.5% to 98.0% identity, depending on the gene family (Supplemental Table S7).

To perform taxonomic profiling, MIDAS aligns reads to the database of 15 universal gene families with HS-BLASTN, discards local alignments that cover <70% of the read or alignments that fail to satisfy the gene-specific species-level percent identity cutoffs, and assigns each uniquely mapped read to a species according to its best hit. MIDAS assigns nonuniquely mapped reads (i.e., identical alignment scores to genes from more than one species) using probabilities estimated from uniquely mapped reads. These mapped reads are used to estimate the coverage and relative abundance of each species.

### Gene content estimation

To estimate gene content, MIDAS first uses the species abundance profile to identify bacterial species with sufficient coverage (e.g., greater than 1×). A pan-genome database is dynamically built, which contains a set of nonredundant genes from these species. We used a 99% sequence identity threshold to cluster similar genes such that any two genes that are <99% similar were classified as distinct genes. Bowtie 2 (Langmead and Salzberg 2012) is used to locally map reads from the metagenome against the pan-genome database. Each read is mapped a single time according to its best hit, and reads with an insufficient mapping percent identity (default = 94%), alignment coverage (default = 70%), mapping quality (default = 20), or sequence quality (default = 20) are discarded.

Mapped reads are used to compute the coverage of the genes clustered at 99% identity. Because the 99% identity may result in many very similar gene families, MIDAS gives the option of further clustering the gene families at lower sequence identities ranging from 75% to 95%. Aggregating enables quantification of gene families of varying size and diversity, while maintaining mapping speed and sensitivity.

To estimate gene copy numbers in a bacterial population, gene coverages are normalized by the median coverage across the 15 universal single-copy gene families (Supplemental Table S7). Copy-number values are thresholded to produce gene presence–absence calls. MIDAS merges these results across multiple metagenomic samples to produce gene content matrices for all species, which facilitate comparative analyses across genes and metagenomic samples.

### Identifying core-genome SNPs

To estimate core-genome SNPs, MIDAS first uses the species abundance profile to identify species with sufficient coverage (e.g., greater than 10×). A representative genome database is dynamically built, which contains a single genome per species that meets the coverage requirement. The representative genome is a single genome chosen that has the greatest nucleotide identity, on average, to other members of the species. Only a single genome is needed for identifying the core genome, because this region should be present in all strains of a species. Bowtie 2 is used to globally map reads to the representative genome database. Each read is mapped a single time according to its best hit, and reads with an insufficient mapping percent identity (default = 94%), alignment coverage (default = 70%), mapping quality (default = 20), or sequence quality (default = 20) are discarded. Additionally, bases with low sequence quality scores are discarded (default = 30). SAMtools (Li et al. 2009) is used to generate a pileup of nucleotides at each genomic position which is parsed to generate output files that report nucleotide variation statistics at all genomic sites. To identify the core genome of a species, MIDAS uses the output from multiple metagenomic samples to identify regions at consistently high coverage (e.g., greater than 10× coverage in 95% of samples) (Supplemental Fig. S6). MIDAS then produces core-genome SNP matrices for all species, which facilitate comparative analyses of nucleotide variation across genomic sites and metagenomic samples. MIDAS also gives the option of outputting all SNPs, including those that are not in the core genome.

### Shotgun simulations and validation of MIDAS output

To validate MIDAS we designed a series of realistic metagenomic simulations using reads from completed genome-sequencing projects deposited in the NCBI Sequence Read Archive (Leinonen et al. 2011) that we identified using the SRAdb tool (Zhu et al. 2013). We used these data to construct 20 mock metagenomes, which each contained 100-bp Illumina reads from 20 randomly selected bacterial genome projects (Supplemental Tables S8, S9). We only selected genome projects that corresponded to one of the 31,007 genomes present in our reference database, and we used only one genome project per species. We simulated libraries that contained 100× total genome coverage. The relative abundances of the 20 genomes were exponentially distributed in each simulation (50%, 25%, 12%, 6.5%, etc.).

We compared the output of MIDAS to the known species abundance, gene content, and SNPs in the simulated communities. To evaluate the accuracy of species abundance estimation, we compared the expected relative abundance and coverage to the simulated relative abundance and coverage. To evaluate the accuracy of gene content estimation, we ran MIDAS to estimate the copy number of genes in the pan-genome of each species in each simulation. We applied a cutoff to these values to predict gene presence–absence. True positives (TP) were present genes predicted as present, false positives (FPs) were absent genes predicted as present, true negatives (TN) were absent genes predicted as absent, and false negatives (FNs) were present genes predicted as absent. Performance was measured across a range of copy-number cutoffs using balanced accuracy: (TPR + TNR)/2, in which TPR = TP/(TP + FN) and TNR = TN/(TN + FP). To evaluate the accuracy of core-genome SNPs, we ran MIDAS to estimate the frequency of nucleotide variants in the representative genome of each species in each simulation. We predicted SNPs using the consensus allele at each genomic position. True SNPs were identified by comparing genomes in the simulations to the representative genomes used for read mapping with the program MUMmer (Kurtz et al. 2004), which identified 3,971,528 total true SNPs. True positives were correctly called SNPs, false positives were incorrectly called SNPs, and false negatives were SNPs that were not called owing to insufficient coverage. We compared predicted SNPs to true SNPs and measured performance using the true positive rate (TP/TP + FN) and precision (TP/TP + FP).

### Assessing database coverage across different environments

We estimated the species-level coverage of the MIDAS database across metagenomes from different environments. Database coverage is defined as the percentage (0%–100%) of genomes from cellular organisms in a community that have a sequenced representative at the species level in the reference database. We estimated database coverage by (1) computing the total coverage across all species in the MIDAS database by mapping metagenomic reads to 15 universal single-copy genes and applying species-level mapping thresholds, (2) computing the coverage across all microbial species, including those absent from the MIDAS reference database using the tool *MicrobeCensus* (Nayfach and Pollard 2015), and (3) taking the ratio of these two quantities, multiplied by 100. We applied this approach to metagenomes from human body sites (The Human Microbiome Project Consortium 2012), human stool (Qin et al. 2012; Li et al. 2014; Obregon-Tito et al. 2015; Rampelli et al. 2015), baboon stool (Tung et al. 2015), mouse stool (Xiao et al. 2015), ocean water (Sunagawa et al. 2015), and soil from deserts, forests, grasslands, and tundra (Fierer et al. 2012). To identify possible taxonomic groups that harbored novel species in the human gut, we performed Spearman correlations between database coverage and the relative abundance of genera in HMP stool samples. Genus-level relative abundances were estimated using mOTU (Sunagawa et al. 2013).

### Tracking transmission of strains between hosts

We used rare SNPs to track transmission of strains between hosts, which we termed “marker alleles.” We defined a marker allele as an allele at a genomic site that was present in only a single individual, or in the case of the mother–infant data set, a single mother–infant pair. For simplicity, we only considered biallelic genomic sites. An allele was determined to be present in a sample if it was supported by three or more reads and ≥10% of the total reads mapped at the genomic site. These parameters were chosen to minimize the effect of sequencing errors and filter out low frequency variants that might not be consistently detected between samples. Marker allele sharing was computed as the percentage of marker alleles in mother strains that were also found in her infant. To minimize variation in marker allele sharing due to sampling, we excluded individuals with fewer than 10 identified marker alleles for a species. We applied this procedure to 66 species found in stool metagenomes from 98 Swedish mothers and their infants (Backhed et al. 2015) and 123 American individuals from the HMP (The Human Microbiome Project Consortium 2012). We included the HMP samples to increase sample sizes and therefore improve the specificity of marker alleles identified in mothers and their infants. As a positive control (i.e., to assess the sensitivity), we quantified marker allele sharing for each species between pairs of technical replicates from the HMP. As a negative control (i.e., to assess specificity), we quantified marker allele sharing for each species between pairs of unrelated individuals from the HMP, which were not used to identify marker alleles. Based on these results, we defined a transmission event as >5% marker allele sharing between a pair of individuals.

### Analysis of globally distributed marine metagenomes

To assess the global population structure of marine bacteria, we analyzed 198 Illumina shotgun metagenomes collected from the *Tara* Oceans expeditions that corresponded to prokaryotic size fractions (SRA Study Accessions: ERP001736, ERP001737) (Supplemental Table S12). We utilized up to 100 million reads per metagenome and analyzed only one sequencing run per sample accession. In cases in which there were multiple sequencing runs per sample accession, we used the sequencing run with the greatest number of reads. We used MIDAS to quantify the relative abundance of the 5952 reference species and, based on these results, identified 30 species that occurred at greater than 3× sequencing depth in the greatest number of metagenomes. The least prevalent species was found in 23% of metagenomes. Next, we used MIDAS to quantify the gene content of these species across metagenomic samples. Reads were mapped to the pan-genome database, and reads with <94% alignment identity were discarded. Mapped reads were used to compute the coverage of genes clustered at 95% identity. Gene coverages were normalized by the coverage of 15 universal-single-copy genes to estimate gene copy numbers. We estimated gene presence–absence by thresholding the gene copy numbers, whereby any gene with a copy number less than 0.35 was considered to be absent.

To uncover population structure, we performed a principal component analysis of the gene presence–absence matrix for each species. To assess the relationship between gene content and geography, we first quantified the PCA distance and geographic distance between metagenomic samples for each species at each water layer. PCA distances were computed using the Euclidian distance between samples based on the first two principal components. Geographic distances were computed using the great-circle distance with the R package geosphere (https://cran.r-project.org/web/packages/geosphere/index.html). Mantel tests were computed using the R package vegan (https://cran.r-project.org/web/packages/vegan/index.html) to correlate the PCA distances to the geographic distances. At each water layer, we only included one metagenome per sampling location and only included species observed at more than five sampling locations. Up to 1 million permutations were performed to assess significance.

### Software availability

MIDAS is implemented in Python and is freely available, along with documentation, at https://github.com/snayfach/MIDAS. Source code is additionally included as Supplemental Material. Our reference database of bacterial species and associated genomic data resources are available at http://lighthouse.ucsf.edu/MIDAS.

## Supplementary Material

Supplemental Material

## References

[NAYFACHGR201863C1] Altschul SF, Gish W, Miller W, Myers EW, Lipman DJ. 1990 Basic local alignment search tool. J Mol Biol 215: 403–410. 2231712 10.1016/S0022-2836(05)80360-2

[NAYFACHGR201863C2] Ashburner M, Ball CA, Blake JA, Botstein D, Butler H, Cherry JM, Davis AP, Dolinski K, Dwight SS, Eppig JT, 2000 Gene ontology: tool for the unification of biology. Nat Genet 25: 25–29. 10802651 10.1038/75556 PMC3037419

[NAYFACHGR201863C3] Backhed F, Roswall J, Peng Y, Feng Q, Jia H, Kovatcheva-Datchary P, Li Y, Xia Y, Xie H, Zhong H, 2015 Dynamics and stabilization of the human gut microbiome during the first year of life. Cell Host Microbe 17: 690–703. 25974306 10.1016/j.chom.2015.04.004

[NAYFACHGR201863C4] Bokulich NA, Chung J, Battaglia T, Henderson N, Jay M, Li H, Lieber AD, Wu F, Perez-Perez GI, Chen Y, 2016 Antibiotics, birth mode, and diet shape microbiome maturation during early life. Sci Transl Med 8: 343ra382. 10.1126/scitranslmed.aad7121 PMC5308924 27306664

[NAYFACHGR201863C5] Browne HP, Forster SC, Anonye BO, Kumar N, Neville BA, Stares MD, Goulding D, Lawley TD. 2016 Culturing of ‘unculturable’ human microbiota reveals novel taxa and extensive sporulation. Nature 533: 543–546. 27144353 10.1038/nature17645 PMC4890681

[NAYFACHGR201863C6] Chen Y, Ye W, Zhang Y, Xu Y. 2015 High speed BLASTN: an accelerated MegaBLAST search tool. Nucleic Acids Res 43: 7762–7768. 26250111 10.1093/nar/gkv784 PMC4652774

[NAYFACHGR201863C7] Cleary B, Brito IL, Huang K, Gevers D, Shea T, Young S, Alm EJ. 2015 Detection of low-abundance bacterial strains in metagenomic datasets by eigengenome partitioning. Nat Biotechnol 33: 1053–1060. 26368049 10.1038/nbt.3329 PMC4720164

[NAYFACHGR201863C8] Eddy SR. 2011 Accelerated profile HMM searches. PLoS Comput Biol 7: e1002195. 22039361 10.1371/journal.pcbi.1002195 PMC3197634

[NAYFACHGR201863C9] Edgar RC. 2010 Search and clustering orders of magnitude faster than BLAST. Bioinformatics 26: 2460–2461. 20709691 10.1093/bioinformatics/btq461

[NAYFACHGR201863C10] Faith JJ, Guruge JL, Charbonneau M, Subramanian S, Seedorf H, Goodman AL, Clemente JC, Knight R, Heath AC, Leibel RL, 2013 The long-term stability of the human gut microbiota. Science 341: 1237439. 23828941 10.1126/science.1237439 PMC3791589

[NAYFACHGR201863C11] Fierer N, Leff JW, Adams BJ, Nielsen UN, Bates ST, Lauber CL, Owens S, Gilbert JA, Wall DH, Caporaso JG. 2012 Cross-biome metagenomic analyses of soil microbial communities and their functional attributes. Proc Natl Acad Sci 109: 21390–21395. 23236140 10.1073/pnas.1215210110 PMC3535587

[NAYFACHGR201863C12] Francis OE, Bendall M, Manimaran S, Hong C, Clement NL, Castro-Nallar E, Snell Q, Schaalje GB, Clement MJ, Crandall KA, 2013 *Pathoscope*: species identification and strain attribution with unassembled sequencing data. Genome Res 23: 1721–1729. 23843222 10.1101/gr.150151.112 PMC3787268

[NAYFACHGR201863C13] Goodrich JK, Davenport ER, Beaumont M, Jackson MA, Knight R, Ober C, Spector TD, Bell JT, Clark AG, Ley RE. 2016 Genetic determinants of the gut microbiome in UK twins. Cell Host Microbe 19: 731–743. 27173935 10.1016/j.chom.2016.04.017 PMC4915943

[NAYFACHGR201863C14] Greenblum S, Carr R, Borenstein E. 2015 Extensive strain-level copy-number variation across human gut microbiome species. Cell 160: 583–594. 25640238 10.1016/j.cell.2014.12.038 PMC4507803

[NAYFACHGR201863C15] The Human Microbiome Project Consortium. 2012 A framework for human microbiome research. Nature 486: 215–221. 22699610 10.1038/nature11209 PMC3377744

[NAYFACHGR201863C16] Kanehisa M, Goto S. 2000 KEGG: Kyoto encyclopedia of genes and genomes. Nucleic Acids Res 28: 27–30. 10592173 10.1093/nar/28.1.27 PMC102409

[NAYFACHGR201863C17] Kashtan N, Roggensack SE, Rodrigue S, Thompson JW, Biller SJ, Coe A, Ding H, Marttinen P, Malmstrom RR, Stocker R, 2014 Single-cell genomics reveals hundreds of coexisting subpopulations in wild *Prochlorococcus*. Science 344: 416–420. 24763590 10.1126/science.1248575

[NAYFACHGR201863C18] Koenig JE, Spora A, Scalfonea N, Frickera AD, Stombaugh J, Knight R, Angenent LT, Ley RE. 2011 Succession of microbial consortia in the developing infant gut microbiome. Proc Natl Acad Sci 108 Suppl 1: 4578–4585. 20668239 10.1073/pnas.1000081107 PMC3063592

[NAYFACHGR201863C19] Konstantinidis KT, Ramette A, Tiedje JM. 2006 The bacterial species definition in the genomic era. Philos Trans R Soc Lond B Biol Sci 361: 1929–1940. 17062412 10.1098/rstb.2006.1920 PMC1764935

[NAYFACHGR201863C20] Kurtz S, Phillippy A, Delcher AL, Smoot M, Shumway M, Antonescu C, Salzberg SL. 2004 Versatile and open software for comparing large genomes. Genome Biol 5: R12. 14759262 10.1186/gb-2004-5-2-r12 PMC395750

[NAYFACHGR201863C21] Ladau J, Sharpton TJ, Finucane MM, Jospin G, Kembel SW, O'Dwyer J, Koeppel AF, Green JL, Pollard KS. 2013 Global marine bacterial diversity peaks at high latitudes in winter. ISME J 7: 1669–1677. 23514781 10.1038/ismej.2013.37 PMC3749493

[NAYFACHGR201863C22] Land M, Hauser L, Jun SR, Nookaew I, Leuze MR, Ahn TH, Karpinets T, Lund O, Kora G, Wassenaar T, 2015 Insights from 20 years of bacterial genome sequencing. Funct Integr Genomics 15: 141–161. 25722247 10.1007/s10142-015-0433-4 PMC4361730

[NAYFACHGR201863C23] Langmead B, Salzberg SL. 2012 Fast gapped-read alignment with Bowtie 2. Nat Methods 9: 357–359. 22388286 10.1038/nmeth.1923 PMC3322381

[NAYFACHGR201863C24] Leinonen R, Sugawara H, Shumway M, International Nucleotide Sequence Database Collaboration. 2011 The sequence read archive. Nucleic Acids Res 39(Database issue): D19–D21. 21062823 10.1093/nar/gkq1019 PMC3013647

[NAYFACHGR201863C25] Li H, Handsaker B, Wysoker A, Fennell T, Ruan J, Homer N, Marth G, Abecasis G, Durbin R, Genome Project Data Processing Subgroup. 2009 The Sequence Alignment/Map format and SAMtools. Bioinformatics 25: 2078–2079. 19505943 10.1093/bioinformatics/btp352 PMC2723002

[NAYFACHGR201863C26] Li J, Jia H, Cai X, Zhong H, Feng Q, Sunagawa S, Arumugam M, Kultima JR, Prifti E, Nielsen T, 2014 An integrated catalog of reference genes in the human gut microbiome. Nat Biotechnol 32: 834–841. 24997786 10.1038/nbt.2942

[NAYFACHGR201863C27] Li SS, Zhu A, Benes V, Costea PI, Hercog R, Hildebrand F, Huerta-Cepas J, Nieuwdorp M, Salojarvi J, Voigt AY, 2016 Durable coexistence of donor and recipient strains after fecal microbiota transplantation. Science 352: 586–589. 27126044 10.1126/science.aad8852

[NAYFACHGR201863C28] Lieberman TD, Flett KB, Yelin I, Martin TR, McAdam AJ, Priebe GP, Kishony R. 2014 Genetic variation of a bacterial pathogen within individuals with cystic fibrosis provides a record of selective pressures. Nat Genet 46: 82–87. 24316980 10.1038/ng.2848 PMC3979468

[NAYFACHGR201863C29] Loewenstein Y, Portugaly E, Fromer M, Linial M. 2008 Efficient algorithms for accurate hierarchical clustering of huge datasets: tackling the entire protein space. Bioinformatics 24: i41–i49. 18586742 10.1093/bioinformatics/btn174 PMC2718652

[NAYFACHGR201863C30] Luo C, Knight R, Siljander H, Knip M, Xavier RJ, Gevers D. 2015 ConStrains identifies microbial strains in metagenomic datasets. Nat Biotechnol 33: 1045–1052. 26344404 10.1038/nbt.3319 PMC4676274

[NAYFACHGR201863C31] Makino H, Kushiro A, Ishikawa E, Kubota H, Gawad A, Sakai T, Oishi K, Martin R, Ben-Amor K, Knol J, 2013 Mother-to-infant transmission of intestinal bifidobacterial strains has an impact on the early development of vaginally delivered infant's microbiota. PLoS One 8: e78331. 24244304 10.1371/journal.pone.0078331 PMC3828338

[NAYFACHGR201863C32] Martín V, Maldonado-Barragán A, Moles L, Rodriguez-Baños M, Campo RD, Fernández L, Rodríguez JM, Jiménez E. 2012 Sharing of bacterial strains between breast milk and infant feces. J Hum Lact 28: 36–44. 22267318 10.1177/0890334411424729

[NAYFACHGR201863C33] Mende DR, Sunagawa S, Zeller G, Bork P. 2013 Accurate and universal delineation of prokaryotic species. Nat Methods 10: 881–884. 23892899 10.1038/nmeth.2575

[NAYFACHGR201863C34] Meyer F, Overbeek R, Rodriguez A. 2009 FIGfams: yet another set of protein families. Nucleic Acids Res 37: 6643–6654. 19762480 10.1093/nar/gkp698 PMC2777423

[NAYFACHGR201863C35] Milani C, Mancabelli L, Lugli GA, Duranti S, Turroni F, Ferrario C, Mangifesta M, Viappiani A, Ferretti P, Gorfer V, 2015 Exploring vertical transmission of bifidobacteria from mother to child. Appl Environ Microbiol 81: 7078–7087. 26231653 10.1128/AEM.02037-15 PMC4579462

[NAYFACHGR201863C36] Nayfach S, Pollard KS. 2015 Average genome size estimation improves comparative metagenomics and sheds light on the functional ecology of the human microbiome. Genome Biol 16: 51. 25853934 10.1186/s13059-015-0611-7 PMC4389708

[NAYFACHGR201863C37] Nielsen HB, Almeida M, Juncker AS, Rasmussen S, Li J, Sunagawa S, Plichta DR, Gautier L, Pedersen AG, Le Chatelier E, 2014 Identification and assembly of genomes and genetic elements in complex metagenomic samples without using reference genomes. Nat Biotechnol 32: 822–828. 24997787 10.1038/nbt.2939

[NAYFACHGR201863C38] Novembre J, Johnson T, Bryc K, Kutalik Z, Boyko AR, Auton A, Indap A, King KS, Bergmann S, Nelson MR, 2008 Genes mirror geography within Europe. Nature 456: 98–101. 18758442 10.1038/nature07331 PMC2735096

[NAYFACHGR201863C39] Obregon-Tito AJ, Tito RY, Metcalf J, Sankaranarayanan K, Clemente JC, Ursell LK, Zech Xu Z, Van Treuren W, Knight R, Gaffney PM, 2015 Subsistence strategies in traditional societies distinguish gut microbiomes. Nat Commun 6: 6505. 25807110 10.1038/ncomms7505 PMC4386023

[NAYFACHGR201863C40] Qin J, Li Y, Cai Z, Li S, Zhu J, Zhang F, Liang S, Zhang W, Guan Y, Shen D, 2012 A metagenome-wide association study of gut microbiota in type 2 diabetes. Nature 490: 55–60. 23023125 10.1038/nature11450

[NAYFACHGR201863C41] Rampelli S, Schnorr SL, Consolandi C, Turroni S, Severgnini M, Peano C, Brigidi P, Crittenden AN, Henry AG, Candela M. 2015 Metagenome sequencing of the Hadza hunter-gatherer gut microbiota. Curr Biol 25: 1682–1693. 25981789 10.1016/j.cub.2015.04.055

[NAYFACHGR201863C42] Rasko DA, Webster DR, Sahl JW, Bashir A, Boisen N, Scheutz F, Paxinos EE, Sebra R, Chin CS, Iliopoulos D, 2011 Origins of the *E. coli* strain causing an outbreak of hemolytic–uremic syndrome in Germany. N Engl J Med 365: 709–717. 21793740 10.1056/NEJMoa1106920 PMC3168948

[NAYFACHGR201863C43] Richter M, Rossello-Mora R. 2009 Shifting the genomic gold standard for the prokaryotic species definition. Proc Natl Acad Sci 106: 19126–19131. 19855009 10.1073/pnas.0906412106 PMC2776425

[NAYFACHGR201863C44] Rinke C, Schwientek P, Sczyrba A, Ivanova NN, Anderson IJ, Cheng JF, Darling A, Malfatti S, Swan BK, Gies EA, 2013 Insights into the phylogeny and coding potential of microbial dark matter. Nature 499: 431–437. 23851394 10.1038/nature12352

[NAYFACHGR201863C45] Rosen MJ, Davison M, Bhaya D, Fisher DS. 2015 Fine-scale diversity and extensive recombination in a quasisexual bacterial population occupying a broad niche. Science 348: 1019–1023. 26023139 10.1126/science.aaa4456

[NAYFACHGR201863C46] Sahl JW, Schupp JM, Rasko DA, Colman RE, Foster JT, Keim P. 2015 Phylogenetically typing bacterial strains from partial SNP genotypes observed from direct sequencing of clinical specimen metagenomic data. Genome Med 7: 52. 26136847 10.1186/s13073-015-0176-9 PMC4487561

[NAYFACHGR201863C47] Schloissnig S, Arumugam M, Sunagawa S, Mitreva M, Tap J, Zhu A, Waller A, Mende DR, Kultima JR, Martin J, 2013 Genomic variation landscape of the human gut microbiome. Nature 493: 45–50. 23222524 10.1038/nature11711 PMC3536929

[NAYFACHGR201863C48] Schnorr SL, Candela M, Rampelli S, Centanni M, Consolandi C, Basaglia G, Turroni S, Biagi E, Peano C, Severgnini M, 2014 Gut microbiome of the Hadza hunter-gatherers. Nat Commun 5: 3654. 24736369 10.1038/ncomms4654 PMC3996546

[NAYFACHGR201863C49] Scholz M, Ward DV, Pasolli E, Tolio T, Zolfo M, Asnicar F, Truong DT, Tett A, Morrow AL, Segata N. 2016 Strain-level microbial epidemiology and population genomics from shotgun metagenomics. Nat Methods 13: 435–438. 26999001 10.1038/nmeth.3802

[NAYFACHGR201863C50] Shapiro BJ, Friedman J, Cordero OX, Preheim SP, Timberlake SC, Szabo G, Polz MF, Alm EJ. 2012 Population genomics of early events in the ecological differentiation of bacteria. Science 336: 48–51. 22491847 10.1126/science.1218198 PMC3337212

[NAYFACHGR201863C51] Snitkin ES, Zelazny AM, Montero CI, Stock F, Mijares L; NISC-Comparative-Sequence-Program, Murray PR, Segre JA. 2011 Genome-wide recombination drives diversification of epidemic strains of *Acinetobacter baumannii*. Proc Natl Acad Sci 108: 13758–13763. 21825119 10.1073/pnas.1104404108 PMC3158218

[NAYFACHGR201863C52] Stepanauskas R. 2012 Single cell genomics: an individual look at microbes. Curr Opin Microbiol 15: 613–620. 23026140 10.1016/j.mib.2012.09.001

[NAYFACHGR201863C53] Sunagawa S, Mende DR, Zeller G, Izquierdo-Carrasco F, Berger SA, Kultima JR, Coelho LP, Arumugam M, Tap J, Nielsen HB, 2013 Metagenomic species profiling using universal phylogenetic marker genes. Nat Methods 10: 1196–1199. 24141494 10.1038/nmeth.2693

[NAYFACHGR201863C54] Sunagawa S, Coelho LP, Chaffron S, Kultima JR, Labadie K, Salazar G, Djahanschiri B, Zeller G, Mende DR, Alberti A, 2015 Ocean plankton. Structure and function of the global ocean microbiome. Science 348: 1261359. 25999513 10.1126/science.1261359

[NAYFACHGR201863C55] Tannock GW, Fuller R, Smith SL, Hall MA. 1990 Plasmid profiling of members of the family *Enterobacteriaceae*, *Lactobacilli*, and *Bifidobacteria* to study the transmission of bacteria from mother to infant. J Clin Microbiol 28: 1225–1228. 2380352 10.1128/jcm.28.6.1225-1228.1990 PMC267909

[NAYFACHGR201863C56] Tu Q, He Z, Zhou J. 2014 Strain/species identification in metagenomes using genome-specific markers. Nucleic Acids Res 42: e67. 24523352 10.1093/nar/gku138 PMC4005670

[NAYFACHGR201863C57] Tung J, Barreiro LB, Burns MB, Grenier JC, Lynch J, Grieneisen LE, Altmann J, Alberts SC, Blekhman R, Archie EA. 2015 Social networks predict gut microbiome composition in wild baboons. eLife 4: e05224. 10.7554/eLife.05224 PMC4379495 25774601

[NAYFACHGR201863C58] Varghese NJ, Mukherjee S, Ivanova N, Konstantinidis KT, Mavrommatis K, Kyrpides NC, Pati A. 2015 Microbial species delineation using whole genome sequences. Nucleic Acids Res 43: 6761–6771. 26150420 10.1093/nar/gkv657 PMC4538840

[NAYFACHGR201863C59] Wattam AR, Abraham D, Dalay O, Disz TL, Driscoll T, Gabbard JL, Gillespie JJ, Gough R, Hix D, Kenyon R, 2014 PATRIC, the bacterial bioinformatics database and analysis resource. Nucleic Acids Res 42(Database issue): D581–D591. 24225323 10.1093/nar/gkt1099 PMC3965095

[NAYFACHGR201863C60] Wu D, Hugenholtz P, Mavromatis K, Pukall R, Dalin E, Ivanova NN, Kunin V, Goodwin L, Wu M, Tindall BJ, 2009 A phylogeny-driven genomic encyclopaedia of Bacteria and Archaea. Nature 462: 1056–1060. 20033048 10.1038/nature08656 PMC3073058

[NAYFACHGR201863C61] Wu D, Jospin G, Eisen JA. 2013 Systematic identification of gene families for use as “markers” for phylogenetic and phylogeny-driven ecological studies of bacteria and archaea and their major subgroups. PLoS One 8: e77033. 24146954 10.1371/journal.pone.0077033 PMC3798382

[NAYFACHGR201863C62] Xiao L, Feng Q, Liang S, Sonne SB, Xia Z, Qiu X, Li X, Long H, Zhang J, Zhang D, 2015 A catalog of the mouse gut metagenome. Nat Biotechnol 33: 1103–1108. 26414350 10.1038/nbt.3353

[NAYFACHGR201863C63] Yassour M, Vatanen T, Siljander H, Hämäläinen AM, Härkönen T, Ryhänen SJ, Franzosa EA, Vlamakis H, Huttenhower C, Gevers D, 2016 Natural history of the infant gut microbiome and impact of antibiotic treatment on bacterial strain diversity and stability. Sci Transl Med 8: 343ra81. 10.1126/scitranslmed.aad0917 PMC5032909 27306663

[NAYFACHGR201863C64] Zhu Y, Stephens RM, Meltzer PS, Davis SR. 2013 SRAdb: query and use public next-generation sequencing data from within R. BMC Bioinformatics 14: 19. 23323543 10.1186/1471-2105-14-19 PMC3560148

[NAYFACHGR201863C65] Zhu A, Sunagawa S, Mende DR, Bork P. 2015 Inter-individual differences in the gene content of human gut bacterial species. Genome Biol 16: 82. 25896518 10.1186/s13059-015-0646-9 PMC4428241

