# Supplementary material for: An integrated metagenomics pipeline for strain profiling reveals novel patterns of bacterial transmission and biogeography
Source: Genome Res. 2016 Nov;26(11):1612–25. doi: 10.1101/gr.201863.115 (PMC5088602; doi:10.1101/gr.201863.115)
Supplement: Supplemental Material [file supp_26_11_1612__index.html]

An integrated metagenomics pipeline for strain profiling reveals novel patterns of bacterial transmission and biogeography — Supplemental Material 

# An integrated metagenomics pipeline for strain profiling reveals novel patterns of bacterial transmission and biogeography

## Supplemental Material

**Files in this Data Supplement:**

- Supplemental\_Figures.pdf
- Supplemental\_Tables.xlsx
- Supplemental\_MIDAS\_1.0.0.zip
