# Supplementary material for: An integrated metagenomics pipeline for strain profiling reveals novel patterns of bacterial transmission and biogeography
Source: Genome Res. 2016 Nov;26(11):1612–25. doi: 10.1101/gr.201863.115 (PMC5088602; doi:10.1101/gr.201863.115)
Supplement: Supplemental Material [file supp_gr.201863.115_Supplemental_Figures.pdf]

# Supplementary Information Nayfach et al. 2016

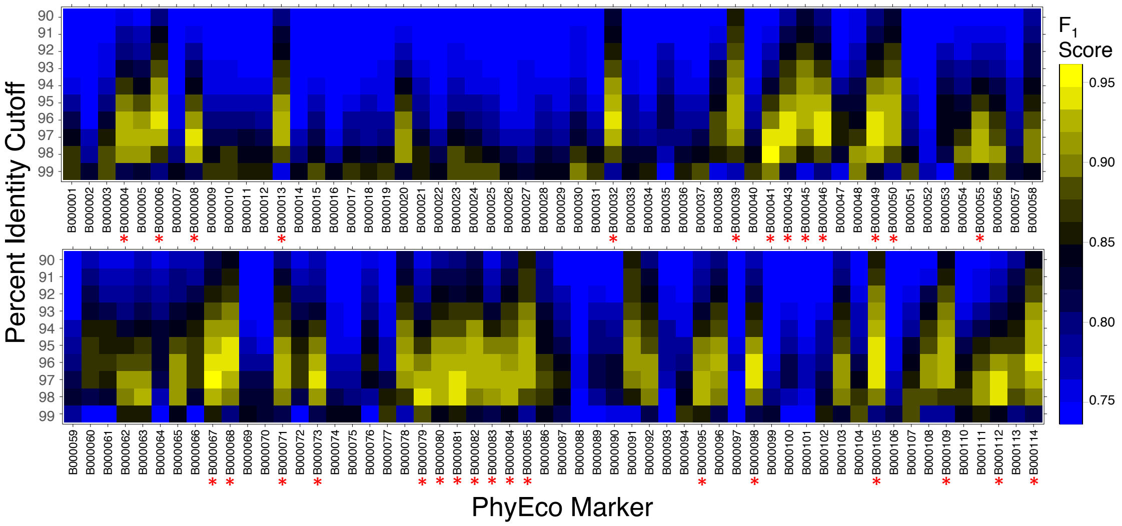


**Supplemental Fig S1. Genome clustering performance for 112 bacterial marker gene families**
PhyEco marker-gene families (Wu et al. 2013) are listed on the horizontal-axis (see also Table S2). The clustering percent identity cutoff is listed on the vertical-axis. Asterisks indicated gene families with the best F1-scores that were selected for genome clustering (Table S2). Cell color indicates the F1-score, which is a measure of clustering performance that balances the true positive rate with precision. True positives were genome pairs with ANI ≥ 95% that were clustered together; false positives were genome pairs with average nucleotide identity (ANI) < 95% that were clustered together; false negatives were genome pairs with ANI ≥ 95% that were assigned to different clusters; and true negatives were genome pairs with ANI < 95% that were assigned to different clusters.

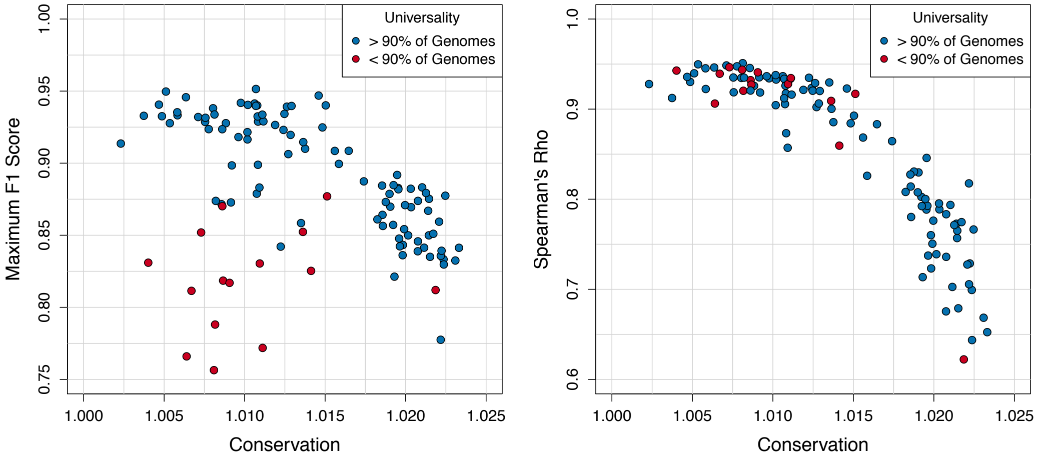


**Supplemental Fig S2. Gene families optimal for genome clustering are conserved and universal.**
Left) Comparison of the conservation and universality of marker genes with clustering performance. Clustering performance measured using the maximum F1-score across percent identity cutoffs (Supplemental table S2). Universality is defined as the proportion of genomes where a gene family is found. Conservation was defined as the average ratio between the marker-gene percent identity ($PID_{i,j}$) and genome wide percent identity ($ANI_i$) across $n$ genome pairs for each marker-gene $j$: $C_j = \frac{1}{n}\sum_i^n \frac{PID_{i,j}}{ANI_i}$. High conservation for a marker-gene indicates low sequence divergence relative to the genomic background. Right) Comparison of the conservation and universality of marker genes with clustering performance defined as the Spearman correlation between marker-gene percent identity and ANI.

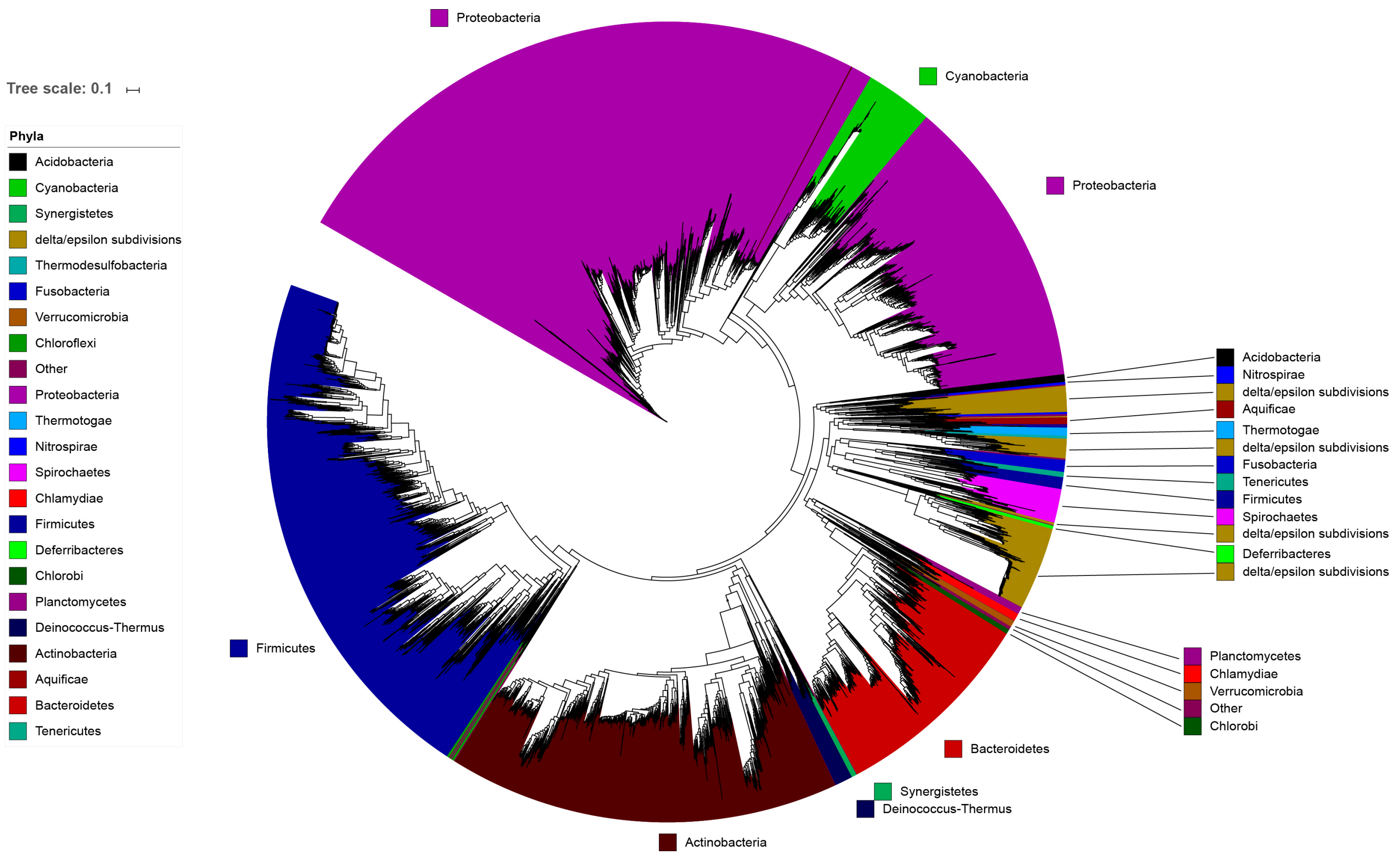


**Supplemental Fig S3. Phylogenetic tree of 5,952 bacterial species.** Maximum likelihood phylogenetic tree of representative genomes from bacterial species using a concatenated protein alignment of the 30 universal gene families used for genome clustering (Supplemental Table S2). Protein sequences of each gene family were first aligned between genomes using MUSCLE (Edgar 2004) (options: -maxiters 2 -diags) and alignment columns with >10% gaps were discarded. Non-discarded alignment columns were concatenated across the 30 genes. FastTree2 (Price et al. 2010) was run with the Jones-Taylor-Thornton (JTT) model of amino acid evolution to construct the phylogenetic tree from the multiple sequence alignment. Tree scale indicates the average number of amino acid substitutions per site. iTOL3 (Letunic and Bork 2016) was used to visualize the tree. Leaves of the tree are annotated by phylum. The tree is available online at: http://lighthouse.ucsf.edu/MIDAS.

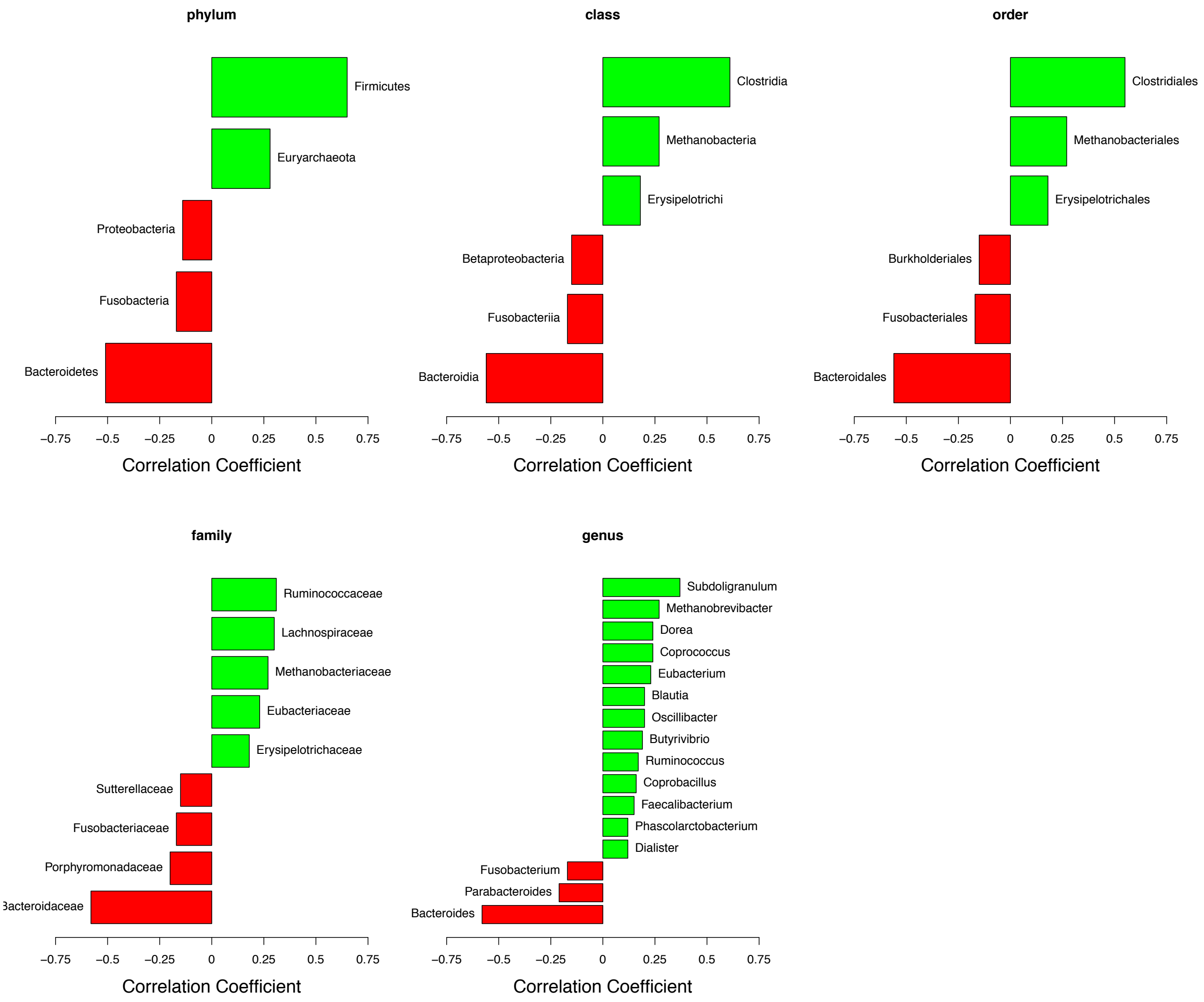


**Supplemental Fig S4. Identification of taxonomic groups correlated with novel species abundance in the human gut.**

The percent of unknown species abundance was estimated for human gut communities from the Human Microbiome Project (The Human Microbiome Project Consortium 2012). This was achieved by computing the coverage of the 5,952 reference species with *MIDAS* and dividing by the total coverage across all microbes obtained with MicrobeCensus. This value was subtracted from 1.0 and represents the fraction of genomes in a community that are novel at the species level. These values were correlated with the relative abundance of taxonomic groups obtained using mOTU (Sunagawa et al. 2013). A positive correlation (green) indicates a taxonomic group that tends to be more abundant in communities with a greater proportion of unknown species. A negative correlation (red) indicates a taxonomic group that tends to be more abundant in communities with a lower proportion of unknown species.

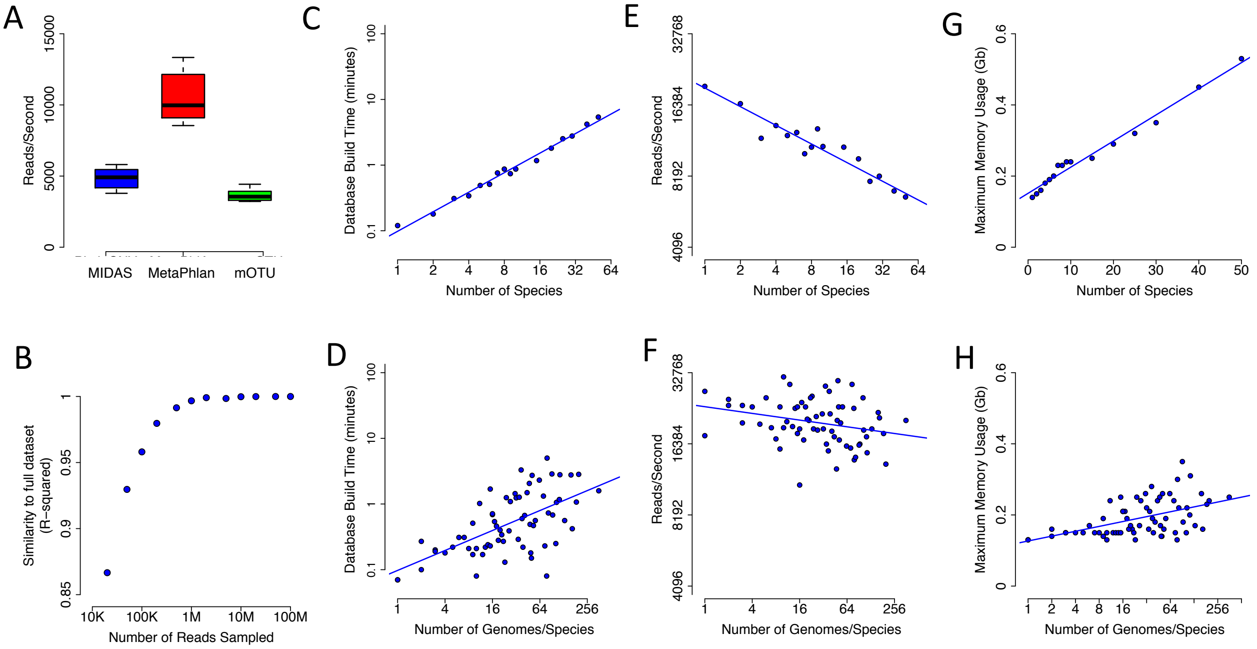


**Supplemental Fig S5. Evaluation of speed and memory usage**

*MIDAS* was evaluated for speed and memory usage with default parameters on 12 metagenomes from the human gut (The Human Microbiome Project Consortium 2012), baboon gut (Tung et al. 2015), ocean (Sunagawa et al. 2015), and soil (Fierer et al. 2012). **A)** Species profiling speed (reads/second) of MIDAS compared to two other widely used methods, MetaPhlAn (Segata et al. 2012) and mOTU (Sunagawa et al. 2013), using default parameters, for ten metagenomes. **B)** *MIDAS* was used to estimate species abundances using between 10K and 100M reads. For each number of reads, the taxonomic profile was compared to the profile estimated using the full dataset. **(C-D)** Database build time using between 1 and 50 species, or between 1 and 256 genomes/species. **(E-F)** Pangenome profiling rate (reads/second), which includes mapping reads to the pan-genome database(s) and computing gene coverages. **(G-H)** Peak memory usage for pan-genome profiling.

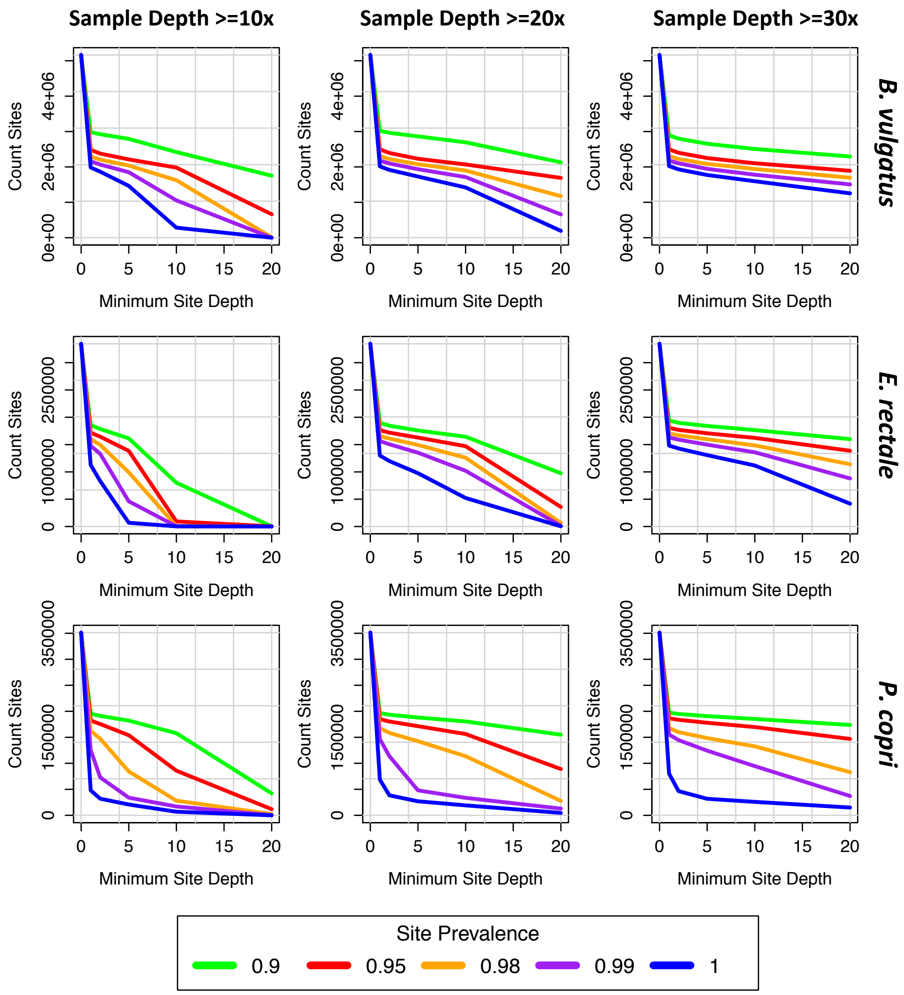


**Supplemental Fig S6. Parameters affecting identification of core-genome sites.**
*MIDAS* combines SNP output files across multiple samples for a given species in order to identify core genomic sites that are at high coverage in all samples. We explored how different options can affect the number of resulting core-genome sites identified for three species: *B. vulgatus*, *E. rectale*, and *P. copri* across stool metagenomes from the Human Microbiome Project. *Sample depth* determines the minimum coverage for a sample to be included. *Minimum site depth* determines the minimum read depth for a site to be included. *Site prevalence* determines the proportion of samples where a site is found at the minimum site depth. We determined cutoffs for these three parameters that produce a sufficient number of high-quality core-genome sites for downstream analyses: site prevalence = 0.95, minimum site depth = 15, and minimum sample depth = 20.

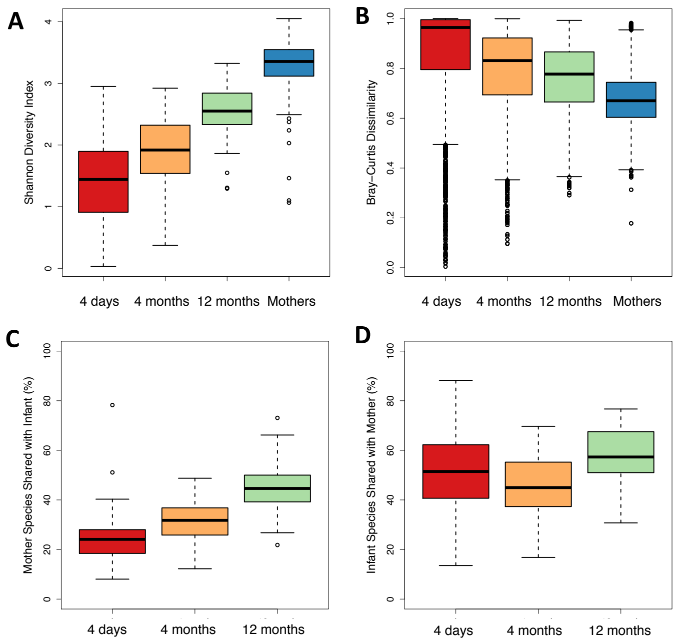


**Supplemental Fig S7. Increase in alpha diversity & decrease in beta diversity over time in the infant gut. A)** Species-level alpha diversity is lowest in the infant gut and increases over time. Alpha diversity was computed using the Shannon diversity index. **B)** Species-level beta diversity is highest in the infant gut at 4 days and decreases over time. Beta diversity was computed using Bray-Curtis dissimilarity between species relative abundance distributions for all pairs of samples from the indicated time point. **(C and D)** The number of shared species between mothers and their infants.

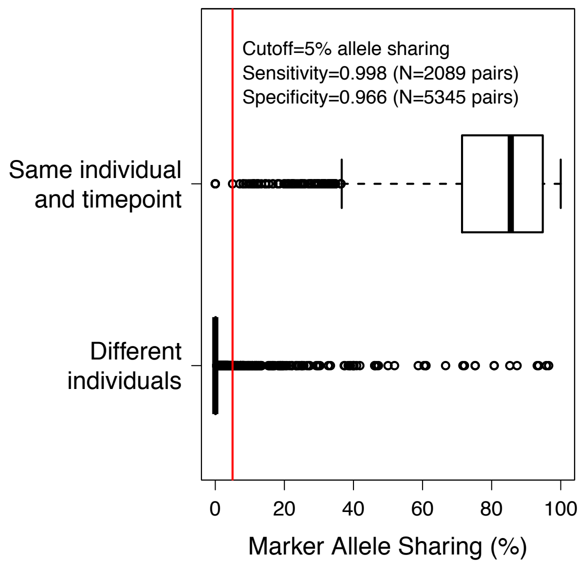


**Supplemental Fig S8. Marker alleles can be used track strains with high sensitivity and specificity.** As a positive control, marker alleles of species were compared between HMP metagenomes from the same individual at the same time point (technical replicates). As a negative control, marker alleles of species were compared between metagenomes from different individuals (non-replicates). A marker allele sharing threshold of 5% clearly separated the positive and negative controls (sensitivity=99.8%, specificity=96.6%).

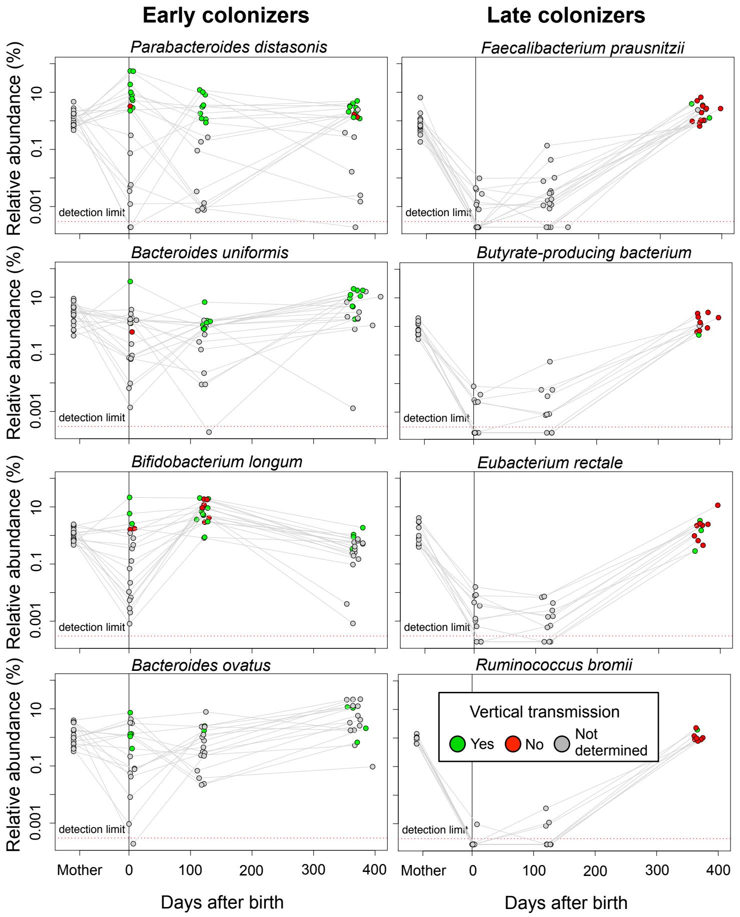


**Supplemental Fig S9. Early and late colonizing species are transmitted from different sources.** Plotted is the relative abundance of early and late colonizing bacterial species in mothers and their infants over the first year of life. Point colors indicate whether the strain of a species was transmitted from an infant's mother (red), not transmitted (green) or whether there was insufficient coverage to call SNPs in an infant and determine transmission (gray). Eight representative examples are shown.

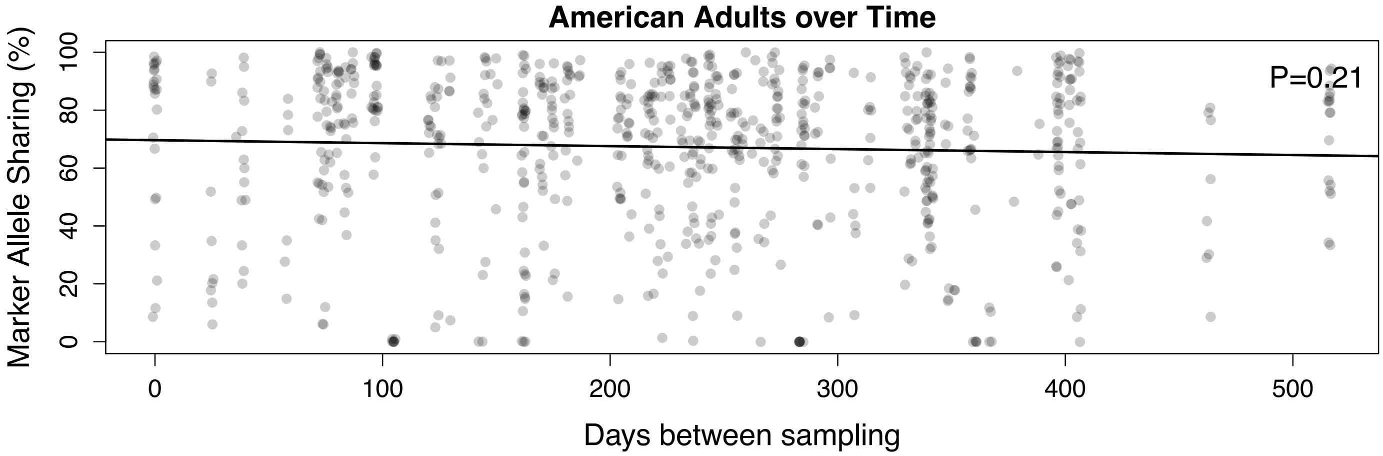


**Supplemental Fig S10. Strains are stable over time in the gut microbiomes of healthy adults.** To evaluate the temporal stability of gut microbiome strains in adults, marker alleles of species were compared between HMP stool metagenomes from the same individual at the different time points. Compared to the infant gut (Figure 3d), marker allele sharing of species found in healthy adults is remarkably stable, indicating that most strains are maintained over time.

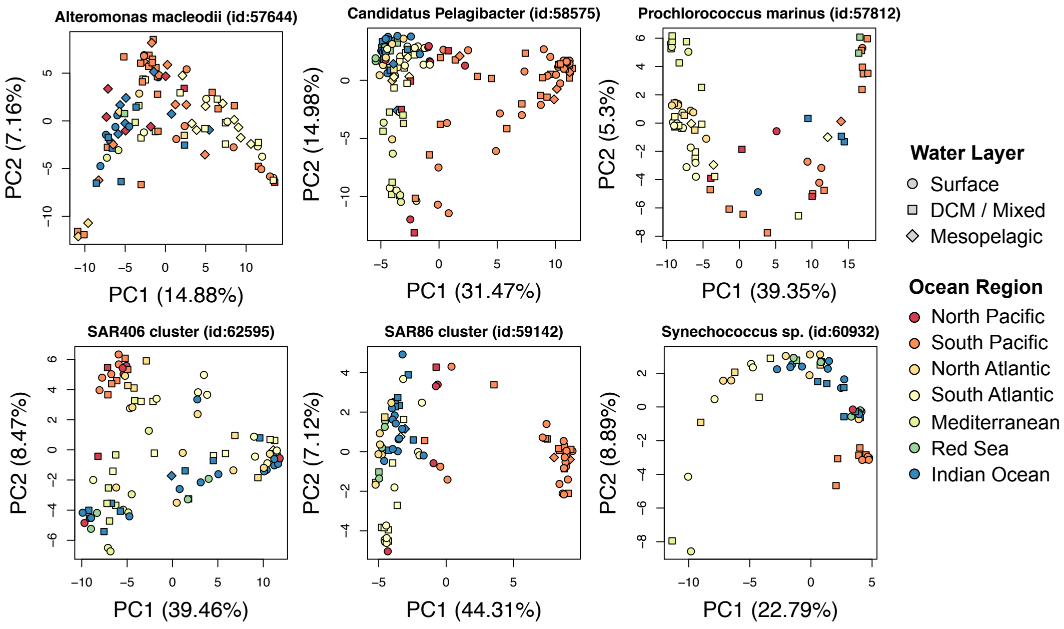


**Supplemental Fig S11. Gene-content based population structure of 6 representative marine species.** Principle component analysis (PCA) was performed for bacterial species based on the presence-absence of gene families. Point colors indicate ocean region and point shape indicates water layer. DCM: deep chlorophyll maximum layer; Mixed: Epipelagic mixed layer.

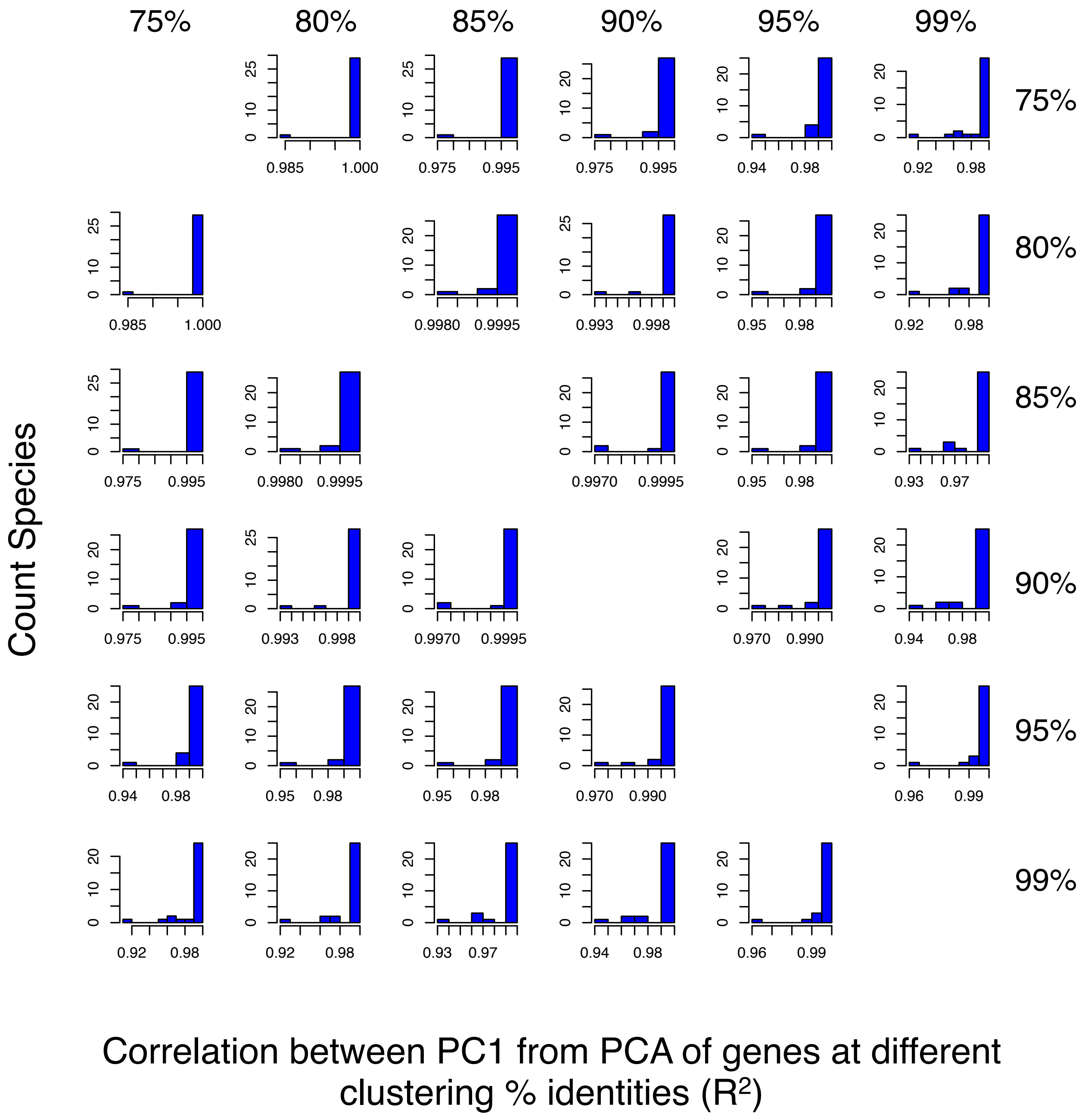


**Supplemental Fig S12. Population structure of marine bacteria based on gene content is robust to the percent identity threshold for defining gene families.** Principle component analysis (PCA) was performed for each of 30 bacterial species based on the presence-absence of gene families. Gene families were defined at 6 different DNA percent identity (%ID) thresholds, listed on the horizontal and vertical axes. For each species, we compared the first principle component (PC1) from PCA performed on gene families at different %ID thresholds. The horizontal axis of each panel indicates the $R^2$ value from this correlation. The vertical axis of each panel indicates the number of species.

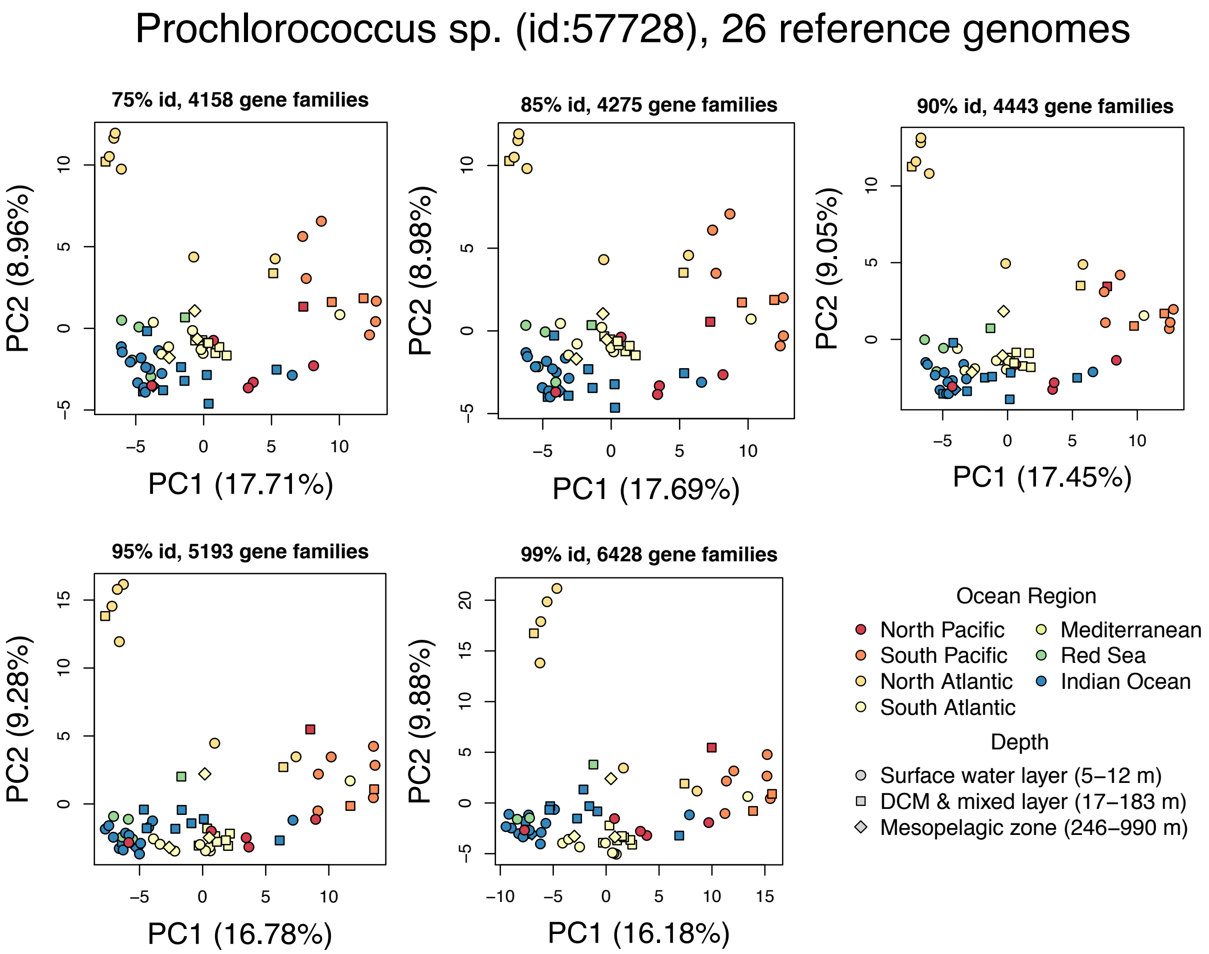


**Supplemental Fig S13. Populations of *Prochlorococcus* have consistent clustering, regardless of the percent identity threshold for defining gene families.** Principle component analysis (PCA) was performed for a large cluster of *Prochlorococcus* genomes (N=26 genomes), based on the presence-absence of gene families. Gene families were defined at different DNA percent identity (%ID) thresholds, listed above each panel. The number of gene families increases with increasing %ID thresholds, but the relationships between the populations, based on gene content PCA, remains constant.

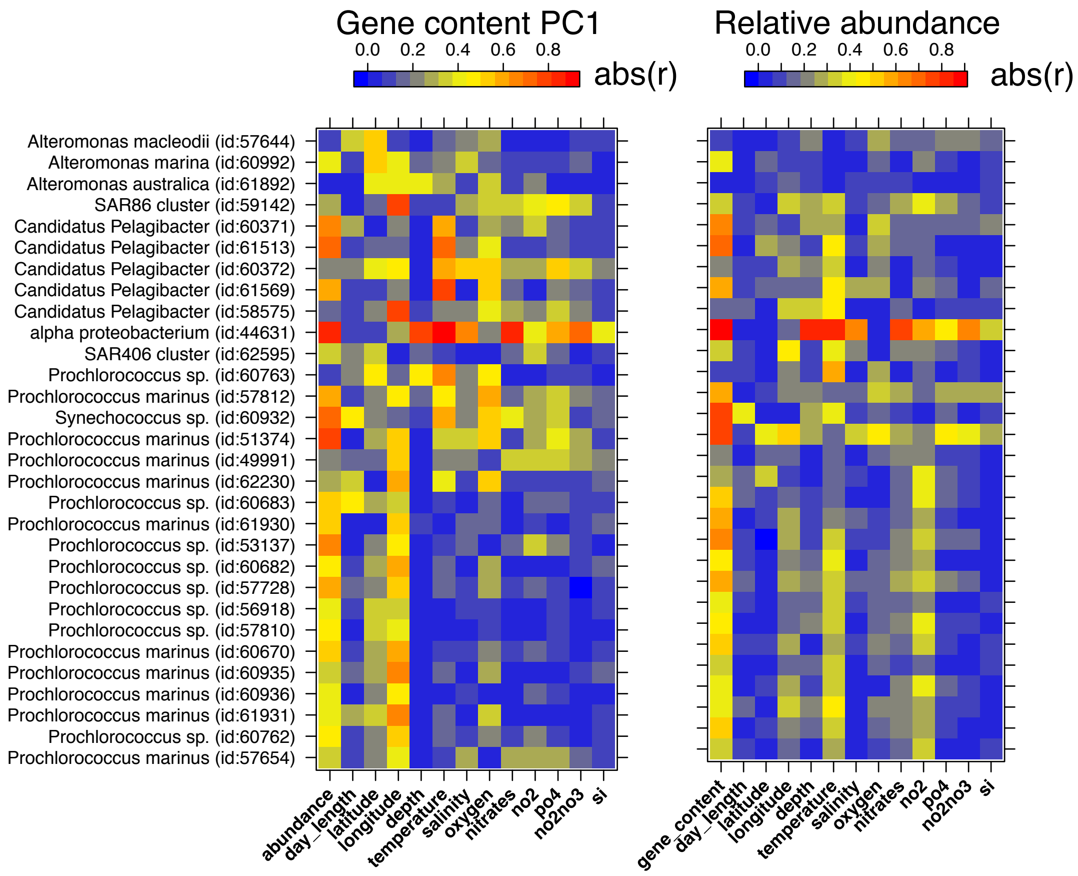


**Supplemental Fig S14. Correlations between gene content, species relative abundance, and environmental parameters in Tara Oceans metagenomes.** Gene content principal component 1 (PC1; left) and species abundance (right) were correlated with environmental parameters (horizontal axis) for different species (vertical axis). For each correlation analysis, the same set of samples was used per species. Heatmap colors reflect the absolute Pearson correlation between variables. Gene content is most strongly correlated with longitude. Species abundance is most strongly correlated with temperature. Neither gene content nor species abundance is strongly correlated with day length, which is a proxy for season. Environmental parameters: abundance = log10(species relative abundance), day_length = average length of day in hours, latitude = latitude of sampling station, longitude = longitude of sampling station, depth = average sampling depth, temperature = average temperature, salinity = average salinity, oxygen = average oxygen concentration, nitrates = mean nitrates concentration, no2 = nitrite concentration, po4 = phosphate concentration, no2no3 = N=nitrite+nitrate concentration, si = silica concentration.

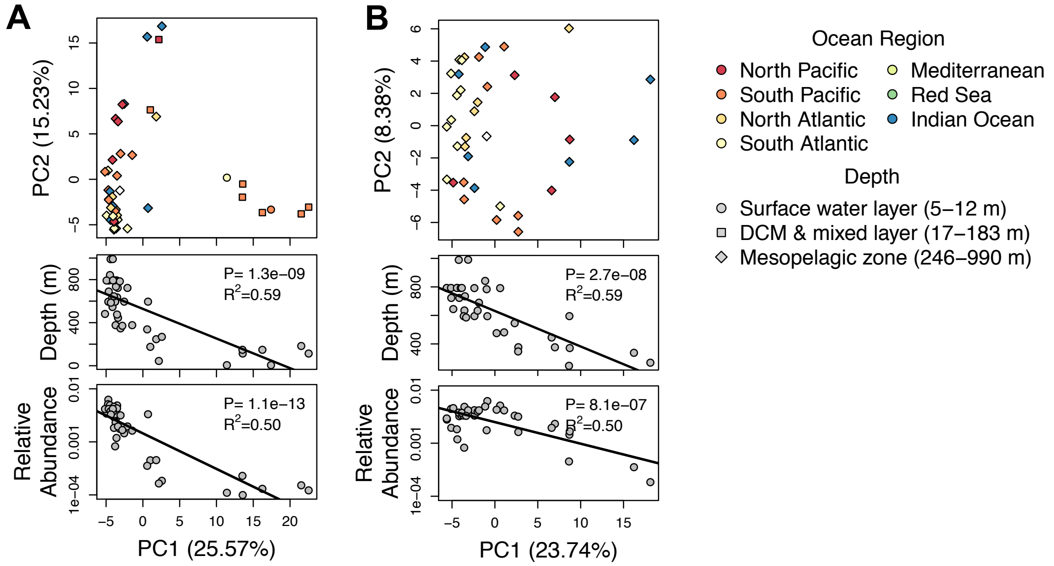


**Supplemental Fig S15. Gene content PCA of alpha proteobacterium (id:44631) with and without samples from the epipelagic zone.** When epipelagic samples are included (left), samples cluster together by water layer based on gene content. When epipelagic samples are excluded (right), there is a clear association with depth below 200m. In both cases, depth and relative abundance are strongly correlated with gene content**.**

# References


Edgar RC. 2004. MUSCLE: multiple sequence alignment with high accuracy and high throughput. *Nucleic Acids Res* **32**(5): 1792-1797.

Fierer N, Leff JW, Adams BJ, Nielsen UN, Bates ST, Lauber CL, Owens S, Gilbert JA, Wall DH, Caporaso JG. 2012. Cross-biome metagenomic analyses of soil microbial communities and their functional attributes. *Proceedings of the National Academy of Sciences*.

Letunic I, Bork P. 2016. Interactive tree of life (iTOL) v3: an online tool for the display and annotation of phylogenetic and other trees. *Nucleic Acids Res* **44**(W1): W242-245.

Price MN, Dehal PS, Arkin APA. 2010. FastTree 2 – Approximately Maximum-Likelihood Trees for Large Alignments. *PloS one* **5**(3).

Segata N, Waldron L, Ballarini A, Narasimhan V, Jousson O, Huttenhower C. 2012. Metagenomic microbial community profiling using unique clade-specific marker genes. *Nature methods* **9**(8): 811-814.

Sunagawa S, Coelho LP, Chaffron S, Kultima JR, Labadie K, Salazar G, Djahanschiri B, Zeller G, Mende DR, Alberti A et al. 2015. Ocean plankton. Structure and function of the global ocean microbiome. *Science* **348**(6237): 1261359.

Sunagawa S, Mende DR, Zeller G, Izquierdo-Carrasco F, Berger SA, Kultima JR, Coelho LP, Arumugam M, Tap J, Nielsen HB et al. 2013. Metagenomic species profiling using universal phylogenetic marker genes. *Nature methods* **10**(12): 1196-1199.

The Human Microbiome Project Consortium. 2012. A framework for human microbiome research. *Nature* **486**(7402): 215-221.

Tung J, Barreiro LB, Burns MB, Grenier JC, Lynch J, Grieneisen LE, Altmann J, Alberts SC, Blekhman R, Archie EA. 2015. Social networks predict gut microbiome composition in wild baboons. *eLife* **4**.

Wu D, Jospin G, Eisen JA. 2013. Systematic identification of gene families for use as "markers" for phylogenetic and phylogeny-driven ecological studies of bacteria and archaea and their major subgroups. *PloS one* **8**(10): e77033.
